# Artificial plastic substrate for tracking and exploring antimicrobial-resistant bacteria transmission pathways in aquatic ecosystems

**DOI:** 10.1016/j.isci.2026.118096

**Published:** 2026-10-30

**Authors:** Ifra Ferheen, Eline Boelee, Filippo Chiudioni, Maria Rosa Loffredo, Laura Mancini, Marco Martuzzi, Marc B. Neumann, Kevin Portune, Sonja Wanke, Stefania Marcheggiani

**Affiliations:** 1 Department of Environment and Primary Prevention, National Institute of Health, 00161 Rome, Italy; 2 Deltares, PO Box 177, MH 2600 Delft, the Netherlands; 3 School of Public Health, Imperial College, London, UK; 4 Basque Centre for Climate Change (BC3), 48940 Leioa, Spain; 5 Ikerbasque, Basque Foundation for Science, Bilbao, Spain

**Keywords:** transitional water, sampling tool, pathogens, antibiotic resistance genes, one health, climate changes

## Abstract

Investigating the persistence and dissemination of antibiotic-resistant bacteria (ARBs) in aquatic ecosystems is challenging because conventional methods require large volumes of water, particularly difficult to collect in regions affected by climate-related stress. This study developed and evaluated artificial plastic substrates (APSs) as an effective sampling tool and assessed the potential role of plastic as a transmission vector for ARBs. Five monthly sampling campaigns were conducted at three sites along the Arrone River. A total of 127 ARBs, including extended-spectrum beta-lactamase (ESBL)-producing Enterobacteriaceae and *Vibrio* spp., were isolated from APSs and raw water (RW) and characterized at the microbiological and molecular levels. APSs recovered more ARBs than RW and captured key antibiotic resistance genes, including class 1 integrons and genes associated with β-lactam, carbapenem, tetracycline, sulfonamide, aminoglycoside, chloramphenicol, and efflux pump-mediated resistance. Overall, APSs proved to be an efficient, cost-effective, reproducible, and minimally invasive tool for monitoring ARBs and ARGs in aquatic ecosystems.

## Highlights

• APSs are a reliable tool for sampling and tracking pathogens in aquatic ecosystems
• Plastic substrates represent emerging ARB reservoirs within One Health approach
• Higher ARG diversity on APSs than in RW samples was demonstrated
• The tool is easy to use and low-cost in aquatic ecosystems under climate stressors

## Graphical abstract

**Figure:**
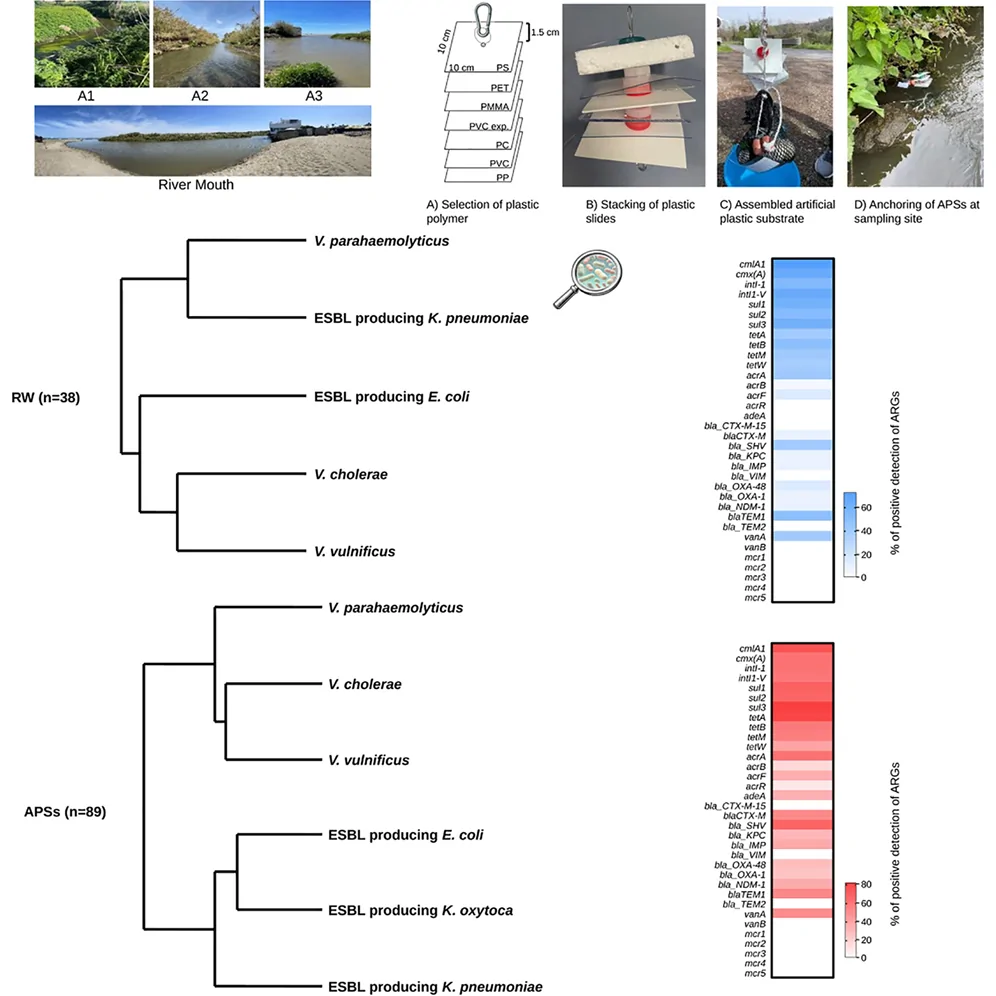

## Teaser

Pathology; Environmental monitoring; Aquatic science

## Introduction

The excessive and inappropriate use of antibiotics in human and veterinary medicine, as well as in agricultural practices, has substantially accelerated the emergence and dissemination of antimicrobial resistance (AMR), thereby compromising the effective management of infectious diseases in both human and animal populations. Antibiotic-resistant bacteria (ARBs) mainly result from genetic mechanisms that occur either through natural genetic variation followed by clonal expansion or via horizontal transfer of mobile genetic elements (MGEs) between bacterial cells (intra- or inter-species).^1^^,^^2^ The emergence of ARBs poses an escalating threat and has severely compromised the effectiveness of infection prevention and treatment, leading to increased morbidity, mortality, and economic burden (WHO, 2021). As infections caused by multidrug-resistant bacteria expand and fewer effective antibiotics are available, forecasts predict that between 2025 and 2050, over 169 million AMR-related deaths could occur worldwide.^3^ While combating AMR in clinical settings remains vital, recent research emphasizes the crucial role of the natural environment in the spread and transmission of ARBs.^4^^,^^5^

Bacteria act as primary colonizers, often autotrophic, and demonstrate a remarkable capacity for structural and metabolic adaptation to challenging environments. They rapidly colonize natural substrates, such as rock surfaces, despite harsh conditions, forming both epilithic and endolithic communities.^6^ In many cases, these communities develop as biofilms, structured multicellular assemblages embedded in an extracellular matrix whose architecture strongly influences resource availability, metabolic activity, survival, and susceptibility to antimicrobials (Schröer et al., 2021^7^). Notably, plastic polymers in the environment can bind, amplify, and disperse potentially pathogenic bacteria, aiding their transfer to new hosts. Due to their hydrophobic surfaces, plastic debris can serve as ideal habitats, providing attachment sites for bacteria and leading to biofilm formation, which transforms plastic debris into a vehicle for pathogenic microorganisms and their associated ARGs.^8^^,^^9^ Microbial biofilms that form on plastic have been termed the “plastisphere”; hence, this living film will shape how these materials interact with their surrounding environments.^10^^,^^11^ Strengthening surveillance systems across the three sectors of the One Health framework, particularly through the Global Antimicrobial Resistance and Use Surveillance System (GLASS), is essential for informing evidence-based public health actions; however, a major challenge remains the generation of high-quality, standardized, and comparable data across sectors.^12^

Investigating the environmental fate of ARBs, pathogenic bacteria, as well as their ARGs, in aquatic ecosystems remains a vital challenge for One Health prevention, as these bacteria are often present at low levels, which makes their detection complex and resource-intensive.^13^ Particularly, their presence in transitional waters such as estuaries and coastal zones poses immediate risks to human, animal, and environmental health.^14^ Transitional waters serve as mixing zones between freshwater and seawater, facilitating the spread of pathogens and AMR genes.^5^ These waters are a primary source of pollution in coastal regions, collecting pollutants and sewage from inland waters and often flowing near beach facilities. Such ecosystems are also common sites for recreational activities, where individuals may unintentionally ingest contaminated water or encounter plastic waste, creating potential exposure pathways for pathogenic bacteria and waterborne diseases.^14^

The sampling method poses the main obstacle to understanding the role of aquatic ecosystems in spreading, because most techniques require collecting and filtering large volumes of water, which can be challenging in regions facing water scarcity.^15^^,^^16^ Additionally, logistical challenges, such as transportation, storage, and processing of these substantial samples, further hinder detection.^13^ Effective management of these pathogenic bacteria requires sensitive, reproducible, and cost-effective methods. Furthermore, understanding the role of emerging contaminants, such as plastic waste, is vital, as they can serve as “hot spots” for bacterial colonization and accumulation, thereby promoting gene exchange.^9^^,^^17^ Not least, aquatic ecosystems are increasingly affected by human activity and climate change, including rising temperatures, water scarcity, flooding, and saline intrusion, posing substantial threats.^18^^,^^19^

This study aims to advance understanding of the role of aquatic ecosystems in the spread of ARBs, pathogenic bacteria and ARGs, within a One Health framework. Specifically, the objectives are to: develop and apply an effective sampling strategy using APSs; and assess their role as potential transmission vectors facilitating the spread of ARBs and their ARGs from inland freshwater systems through transitional waters to the marine environment.

## Results

Site selection was based on a comprehensive land-use analysis conducted in ArcGIS 10.8 (ESRI). The analysis showed that the Arrone River’s 87 km^2^ hydrological catchment comprises 38% agricultural land (predominantly olive groves and vineyards), 29% urbanized areas (including Fiumicino Municipality), and 33% natural vegetation (mainly Mediterranean maquis). Three sampling sites along the Arrone River captured distinct ecohydrological zones: A1, draining volcanic substrates in the upper catchment with minimal anthropogenic influence; A2, in the agriculturally dominated mid-reach receiving runoff from 23 km^2^ of intensively cultivated slopes; and A3, the river mouth, the tidal-influenced lower estuary, which accumulates contaminants from the entire catchment before discharging into the Tyrrhenian Sea (Figure 2A).

### Targeted pathogenic bacteria recovered

Microbiological investigations of both sample types, raw water (RW) and APSs, revealed 127 biochemically identified strains of targeted pathogenic bacteria, of which 70% were isolated from APS samples and 30% from RW. Both extended-spectrum beta-lactamase (ESBL)-producing Enterobacteriaceae and *Vibrio* spp. were recovered at all sites studied. In the APS sample type, the main strains recovered were ESBL-producing *K. pneumoniae* (28.0%), *V. parahaemolyticus* (21.3%), ESBL-producing *E. coli* (19.1%), *V. cholerae* (16.8%), *V. vulnificus* (11.2%), and ESBL-producing *Klebsiella oxytoca* (3.3%) (Figure 2B). In the RW sample type, the main strains recovered were *Vibrio cholerae* (29.0%), *Vibrio parahaemolyticus*, ESBL-producing *E. coli* (23.6% each), ESBL-producing *Klebsiella pneumoniae* (21%), and *Vibrio vulnificus* (2.6%) (Figure 2B). In fact, the boxplot shows a clear difference in both the central tendency and the variability of the number of isolates between the two sample types, and the *t* test revealed a significant difference (*t* = 2.43, *p* ≤ 0.05). The APSs plot shows a higher median of around 20, while the RW plot shows a lower median of approximately 10. A wider interquartile range (IQR) in the APS plot indicates greater variability and dispersion in the data (Figure 2C). Paired *t* tests comparing results from the two sampling methods at each site showed significantly higher recovery with the APSs tool (*p* value ≤ 0.01 for all comparisons), as shown in Figure 2D. Not least, across the three sampling sites, no spatial distribution of bacterial isolates was observed, as all *t* test comparisons yielded *p*-values ≥ 0.05. By contrast, temporal variation was evident, with higher prevalence in June, July, and August than in May and September for both sample types (Figure 2E). In fact, water temperature recorded in the field rose from 18°C in May to a maximum of 26°C in July and August, then declined to 22°C in September. The elevated temperatures coincided with increased prevalence of ARBs in July and August, suggesting a temperature-associated seasonal pattern. Moreover, repeated-measures ANOVA also revealed significant temporal variation across months (F (4,8) = 17.39, *p* values < 0.001), while spatial variation among sites was significant only for APSs (F (2,8) = 5.62, *p* values = 0.032) but not for RW samples (F (2,8) = 2.42, *p* values = 0.15).

Furthermore, negative controls for each culture medium and sampling point exhibited no microbial growth, confirming that no contamination occurred during the experimental procedures.

### Phenotypic antimicrobial susceptibility and ARGs profiles

All 127 strains were multidrug-resistant to the following classes of antibiotics: aminoglycosides (gentamicin [GEN] and kanamycin [KAN]), fluoroquinolones (ciprofloxacin [CIP]), β-lactams (ceftazidime [CAZ], cefoxitin [FOX], and imipenem [IMP]), tetracycline (TET), quinolones (nalidixic acid [NA]), chloramphenicol (CAM), macrolides (erythromycin [ERY]), and sulfonamides (sulfamethoxazole [SMX]) (Tables 1 and 2).

**Table 1:** Phenotypic antimicrobial resistance of pathogenic bacterial species isolated from raw water samples

Phenotypic resistance of RW isolates											
RW associated ARBs	Panel of tested antibiotics^a^										
	CAM (30 μg)	CIP (5 μg)	GEN (10 μg)	ERY (15 μg)	TET (30 μg)	CAZ (30 μg)	KANA (30 μg)	SMX (25 μg)	FOX (30 μg)	IMP (10 μg)	NA (10 μg)
*V. parahemolyticus* (*n* = 9)	R (67%)	R (89%)	R (67%)	R (78%)	R (78%)	R (44%)	R (67%)	R (56%)	R (44%)	S (0%)	R (67%)
*V. cholerae* (*n* = 11)	R (45%)	R (82%)	R (36%)	R (82%)	R (73%)	R (45%)	R (100%)	R (91%)	R (64%)	S (0%)	R (55%)
*V. vulnificus* (*n* = 1)	R (100%)	R (100%)	R (100%)	R (0%)	R (0%)	R (100%)	R (100%)	R 100%)	R (0%)	S (0%)	R (100%)
ESBL-*producing* *K. pneumoniae* (*n* = 8)	R (80%)	R (80%)	R (100%)	R (40%)	R (40%)	R (80%)	R (80%)	R (100%)	R (80%)	S (25%)	R (80%)
ESBL-producing *E. coli* (*n* = 9)	R (75%)	R (50%)	R (100%)	R (50%)	R (100%)	R (100%)	R (100%)	R (75%)	R (100%)	S (0%)	R (100%)

a R, resistance; I, intermediate; S, sensitive.

**Table 2:** Phenotypic antimicrobial resistance of pathogenic bacterial species isolated from APSs samples

APSs associated ARBs	Panel of tested antibiotics^b^										
	CAM (30 μg)	CIP (5 μg)	GEN (10 μg)	ERY (15 μg)	TET (30 μg)	CAZ (30 μg)	KANA (30 μg)	SMX (25 μg)	FOX (30 μg)	IMP (10 μg)	NA (10 μg)
*V. parahemolyticus* (*n* = 19)	R (94%)	R (89%)	R (100%)	R (78%)	R (84%)	R (63%)	R (89%)	R (89%)	R (78%)	S (0%)	R (94%)
*V. cholerae* (*n* = 15)	R (75%)	R (93%)	R (93%)	R (75%)	R (93%)	R (56%)	R (81%)	R (93%)	R (87%)	S (0%)	R (81%)
*V. vulnificus* (*n* = 10)	R (90%)	R (90%)	R (90%)	R (80%)	R (80%)	R (80%)	R (90%)	R (80%)	R (90%)	S (0%)	R (90%)
ESBL-producing *K. pneumoniae* (*n* = 25)	R (80%)	R (92%)	R (100%)	R (80%)	R (92%)	R (96%)	R (92%)	R (96%)	R (84%)	R (73%)	R (84%)
ESBL-producing *E. coli* (*n* = 17)	R (88%)	R (88%)	R (94%)	R (88%)	R (83%)	R (72%)	R (83%)	R (83%)	R (77%)	R (88%)	R (83%)
ESBL-producing *K. oxytoca* (*n* = 3)	R (100%)	R (100%)	R (100%)	R (100%)	R (100%)	R (100%)	R (100%)	R (100%)	R (66%)	R (100%)	R (100%)

b R, resistance; I, intermediate; S, sensitive.

In particular, our Enterobacteriaceae strains exhibited resistance (R) to nearly all antibiotics tested, regardless of whether they were isolated from APSs or RW samples. Only *Vibrio* spp. showed sensitivity (S) to carbapenems. ESBL-producing Enterobacteriaceae strains exhibited high multiple antibiotic resistance (MAR) indices of 0.6–1.0, with more than 90% of isolates recording MAR indices ≥ 0.7. Meanwhile, *Vibrio* spp. showed lower MAR indices, with values ≥0.55 (Tables 3 and 4). The interpretation of these values indicates high-risk environments, depicting strains that are isolated under intense antibiotic pressure.

**Table 3:** Complete characterization of the RW isolates, molecular identification, multiple antimicrobial resistance index, and genotypic Profile

RW strains identification and accession number	MAR index (n°R/total tested antibiotics)^a^	Antibiotic resistance genes - ARGs							
		Beta lactamases	Sulfonamides	Chloramphenicol	Tetracyclines	Glycopeptides	Colistin resistance genes	Efflux pump	Class 1 integrons
*V. parahemolyticus* *PX251262*	0.63 7/11R	negative	*sul1*, *sul3*	*cmlA1*, *cmx(A)*	*tetA*	negative	negative	negative	*intl-1*, *intl1-V*
*V. parahemolyticus* *PX251263*	0.72 8/11R	negative	*sul1*, *sul2*, *sul3*	negative	*tetA*	negative	negative	egative	*intl1-V*
*V. parahemolyticus* *PX251264*	0.63 7/11R	*bla*_TEM_	negative	*cmlA1*	negative	negative	negative	negative	*intl-1*
*V. parahemolyticus* *PX251265*	0.63 7/11R	*bla*_SHV_, *bla*_TEM-1_	*sul1*, *sul3*	*cmlA*, *cmx(A)*	*tetB*	negative	negative	negative	*intl-1*
*V. parahemolyticus* *PX251266*	0.54 6/11R	negative	*sul3*	negative	negative	negative	negative	negative	negative
*V. cholerae* *PX251247*	0.72 8/11R	negative	*sul1*, *sul2*	*cmlA1*, *cmx(A)*	*tetB*, *tetM*	negative	negative	*acrA*	*intl1-V*
*V. cholerae* *PX251248*	0.63 7/11R	*bla*_SHV_, *bla*_TEM-1_	*sul2*, *sul3*	*cmlA1*, *cmx(A)*	*tetA*, *tetM*	negative	negative	*acrA*	*intl-1*
*V. cholerae* *PX251249*	0.63 7/11	negative	*sul1*, *sul2*	*cmlA1*	*tetB*, *tetM*, *tetW*	negative	negative	*acrA*	*intl1-V*
*V. cholerae* *PX251250*	0.54 6/11R	*bla*_TEM-1_	*sul1*	*cmx(A)*	*tetA*, *tetB*	negative	negative	negative	*intl1-V*
*V. cholerae* *PX251251*	0.72 8/11R	*bla*_SHV_	*Sul3*	*cmlA1*, *cmx(A)*	*tetM*, *tetW*	negative	negative	*acrA*	*intl-1*, *intl1-V*
*V. cholerae* *PX251252*	0.63 7/11R	negative	*sul1*, *sul2*, *sul3*	*cmlA1*, *cmx(A)*	*tetM*, *tetW*	negative	negative	*acrA*	*intl1-V*
*V. vulnificus* *PX251241*	0.81 9/11R	*bla*_SHV_, *bla*_TEM-1_	*sul1*, *sul2*, *sul3*	*cmlA1*, *cmx(A)*	*tetA*, *tetB*, *tetM*, *tetW*	negative	negative	*acrA*	*intl-1*, *intl1-V*
*ESBL-producing* *K. pneumoniae* *PX251746*	0.54 6/11R	negative	*sul3*	negative	negative	vanA	negative	negative	negative
ESBL*-producing* *K. pneumoniae* *PX251747*	0.81 9/11R	negative	negative	*cmlA1*	negative	vanA	negative	negative	negative
ESBL*-producing* *K. pneumoniae* *PX251748*	0.63 7/11R	*bla*_OXA-48_	*sul3*	*cmx(A)*	negative	vanA	negative	negative	negative
ESBL*-producing* *K. pneumoniae* *PX251749*	0.63 7/11R	*bla*_NDM-1_	negative	negative	negative	vanA	negative	negative	negative
*ESBL-producing* *E. coli* PX251763	0.54 6/11R	*bla*_CTX-M_, *bla*_KPC_, *bla*_IMP_, *bla*_OXA-1_	*sul1*, *sul2*	*cmlA1*, *cmx(A)*	*tetA*, *tetW*	vanA	negative	*acrA*, *acrF*	*intl-1*, *intl1-V*
ESBL*-producing* *E. coli* PX251764	0.63 7/11R	*bla*_SHV_, *bla*_KPC_, *bla*_NDM-1_, *bla*_TEM-1_	*sul3*	*cmlA1*, *cmx(A)*	*tetB*	vanA	negative	*acrB*, *acrF*	*intl-1*, *intl1-V*
ESBL*-producing* *E. coli* PX251765	0.63 7/11R	*bla*_*IMP*_, *bla*_*OXA-48*_, *bla*_*OXA-1*_	*sul1*, *sul2*, *sul3*	*cmlA1*, *cmx(A)*	*tetB*, *tetM*	vanA	negative	negative	*intl-1*, *intl1-V*
ESBL*-producing* *E. coli* PX251766	0.72 8/11R	*bla*_CTX-M_, *bla*_OXA-48_, *bla*_TEM-1_	*sul1*	*cmlA1*	*tetB*	vanA	negative	*acrF*	*intl1-V*

a Results of phenotypic resistance profiling of species expressed as a multiple antibiotic resistance index (MAR index).

**Table 4:** Complete characterization of the APSs strains molecular identification, multiple antimicrobial resistance index, and genotypic resistance Profile

APSs strains identification and accession number	MAR index (n°R/total tested antibiotics)^b^	Antibiotic resistance genes							
		Beta lactamases	Sulfonamides	Chloramphenicol	Tetracyclines	Glycopeptides	Colistin resistance genes	Efflux pump	Class 1 integrons
*V. parahemolyticus* PX251267	0.81 9/11R	*bla*_CTX-M_, *bla*_SHV_	*sul1*, *sul2*, *sul3*	*cmlA1*	*tetA*, *tetB*, *tetM*	negative	negative	*acrA*, *acrB*, *acrF*, *adeA*	*intl1-V*
*V. parahemolyticus* PX251268	0.72 8/11R	*bla*_SHV_, *bla-*_*TEM1*_	*sul2*, *sul3*	*cmx(A)*	*tetA*, *tetB*, *tetW*	negative	negative	*acrA*, *acrF*	*intl-1*, *intl1-V*
*V. parahemolyticus* PX251269	0.81 9/11R	*bla-*_*TEM1*_	*sul1*, *sul3*	*cmlA1*	*tetA*, *tetM*, *tetW*	negative	negative	*acrA*, *acrR*, *adeA*	*intl1-V*
*V. parahemolyticus* PX251270	0.72 8/11R	*bla-*_*CTXM*_, *bla*_SHV_	*sul1*, *sul2*, *sul3*	*cmlA1*, *cmx(A)*	*tetA*, *tetB*, *tetM*	negative	negative	*acrB*, *acrF*, *adeA*	*intl-1*, *intl1-V*
*V. parahemolyticus* PX251271	0.72 8/11R	*bla*_TEM-1_	*sul1*	negative	*tetA*, *tetB*, *tetM*	negative	negative	*acrA*, *acrR*, *adeA*	negative
*V. parahemolyticus* PX251272	0.81 9/11R	*bla*_CTX-M_	*sul1*, *sul2*, *sul3*	*cmlA1*	*tetA*, *tetB*, *tetM*	negative	negative	*acrA*, *acrB*, *acrF*	*intl1-V*
*V. parahemolyticus* PX251273	0.81 9/11R	*bla*_SHV_	*sul2*, *sul3*	negative	*tetA*, *tetM*, *tetW*	negative	negative	*acrA*, *acrF*, *acrR*	*intl-1*
*V. parahemolyticus* PX251274	0.72 8/11R	*bla*_CTX-M_, *bla*_SHV_	*sul1*, *sul3*	*cmlA1*, *cmx(A)*	*tetB*, *tetM*, *tetW*	negative	negative	*acrB*, *acrF adeA*	*intl1-V*
*V. parahemolyticus* PX251275	0.72 8/11R	*bla*_SHV_, *bla*_TEM-1_	*sul3*	*cmx(A)*	*tetA*, *tetB tetM*	negative	negative	*acrA*, *acrB*, *acrF*	*intl1-V*
*V. cholerae* *PX251259*	0.81 9/11R	*bla*_TEM-1_	*sul1*, *sul2*, *sul3*	*cmlA1*	*tetA*, *tetB*, *tetM*	negative	negative	negative	*intl-1*, *intl1-V*
*V. cholerae* *PV785470*	0.90 10/11R	*bla*_CTX-M_, *bla*_TEM_,	*sul2*, *sul3*	*cmlA1*, *cmx(A)*	*tetA*, *tetB*, *tetM*, *tetW*	negative	negative	negative	*intl-1*, *intl1-V*
*V. cholerae* *PX251253*	0.90 10/11R	*bla*_TEM-1_	*sul1*, *sul2*, *sul3*	*cmlA1*, *cmx(A)*	*tetA*, *tetB*, *tetM*	negative	negative	*acrA*	*intl-1*, *intl1-V*
*V. cholerae* *PX251254*	0.72 8/11R	*bla*_CTX-M_	*sul1*, *sul2*, *sul3*	*cmlA1*	*tetA*, *tetB*, *tetM*, *tetW*	negative	negative	negative	*intl1-V*
*V. cholerae* *PX251255*	0.81 9/11R	negative	*sul1*, *sul2*, *sul3*	*cmlA1*, *cmx(A)*	*tetB*, *tetW*	negative	negative	*acrA*	*intl-1*
*V. cholerae* *PX251256*	0.81 9/11R	*bla*_CTX-M_	*sul1*, *sul3*	*cmx(A)*	*tetA*, *tetM*	negative	negative	negative	*intl-1*, *intl1-V*
*V. cholerae* *PX251257*	0.81 9/11R	*bla*_*TEM-1*_	*sul1*, *sul2*, *sul3*	*cmlA1*, *cmx(A)*	*tetB*, *tetM*	negative	negative	*acrA*	*intl-1*
*V. cholerae* *PX251258*	0.90 10/11R	*bla*_SHV_	*sul1*, *sul2*, *sul3*	*cmlA1*, *cmx(A)*	*tetA*, *tetB*, *tetW*	negative	negative	*acrA*	*intl-1*
*V. vulnificus* PX251242	0.90 10/11R	*bla*_SHV_, *bla*_TEM-1_	*sul1*	*cmlA1*	*tetA*, *tetW*	negative	negative	*acrA*	*intl-1*
*V. vulnificus* PX251243	0.90 10/11R	*bla*_SHV_, *bla*_TEM-1_	*sul2*, *sul3*	*cmlA1*, *cmx(A)*	*tetA*, *tetB*	negative	negative	*acrA*	*intl-1*, *intl1-V*
*V. vulnificus* PX251244	0.90 10/11R	*bla*_CTX-M_, *bla*_SHV_	*sul1*, *sul2*, *sul3*	*cmlA1*, *cmx(A)*	*tetA*, *tetB*, *tetM*	negative	negative	*acrA*	*intl-1*
*V. vulnificus* PX251245	0.81 9/11R	*bla*_CTX-M_, *bla*_TEM-1_	*sul2*, *sul3*	*cmlA1*, *cmx(A)*	*tetA*, *tetM*	negative	negative	*acrA*	*intl-1*, *intl1-V*
*V. vulnificus* PX251246	0.81 9/11R	*bla*_*TEM-1*_	*sul1*	*cmx(A)*	*tetA*, *tetB*	negative	negative	*acrA*	*intl-1*, *intl1-V*
ESBL*-producing K. oxytoca* *PX251743*	1.0 11/11R	*bla*_SHV_, *bla*_KPC_, *bla*_IMP_, *bla*_OXA-48_, *bla*_NDM-1_, *bla*_TEM-1_	*sul1*, *sul2*, *sul3*	*cmlA1*	*tetA*	vanA	negative	*acrA*, *adeA*	*intl-1*
ESBL*-producing K. oxytoca* PX251744	0.90 10/11R	*bla*_CTX-M_, *bla*_SHV_, *bla*_IMP_, *bla*_NDM-1_, *bla*_TEM-1_	*sul1*, *sul2*, *sul3*	*cmlA1*, *cmx(A)*	*tetW*	vanA	negative	*acrA*, *acrF*	*intl-1*, *intl1-V*
ESBL*-producing K. oxytoca* PX251745	1.0 11/11R	*bla*_CTX-M_, *bla*_SHV_, *bla*_KPC_, *bla*_IMP_, *bla*_OXA-48_, *bla*_OXA-1_, *bla*_NDM-1_	*sul1*, *sul2*, *sul3*	*cmlA1*, *cmx(A)*	*tetA*, *tetB*, *tetM*	vanA	negative	*adeA*,	*intl-1*
ESBL*-producing K. pneumoniae* PX251750	0.90 10/11R	*bla*_CTX-M_, *bla*_SHV_, *bla*_KPC_, *bla*_IMP_, *bla*_OXA-48_, *bla*_OXA-1_	*sul3*	*cmlA1*, *cmx(A)*	*tetA*, *tetM*, *tetW*	vanA	negative	*adeA*	*intl1-V*
ESBL*-producing K. pneumoniae* PX251751	1.0 11/11R	*bla*_SHV_, *bla*_KPC_, *bla*_IMP_, *bla*_NDM-1_, *bla*_TEM-1_	*sul1*, *sul2*, *sul3*	*cmx(A)*	*tetA*, *tetB*, *tetM*	vanA	negative	negative	*intl-1*
ESBL*-producing K. pneumoniae* PX251752	0.90 10/11R	*bla*_CTX-M_, *bla*_KPC_, *bla*_OXA-48_, *bla*_*OXA-1*_	*sul2*, *sul3*	*cmlA1*, *cmx(A)*	*tetA*, *tetB*, *tetW*	vanA	negative	negative	*intl1-V*
ESBL*-producing K. pneumoniae* PX251753	0.81 9/11R	*bla*_SHV_, *bla*_KPC_, *bla*_OXA-48_, *bla*_OXA-1_	*sul1*, *sul3*	*cmlA1*	*tetB*, *tetM*	vanA	negative	*adeA*	*intl-1*
ESBL*-producing K. pneumoniae* PX251754	0.81 9/11R	*bla*_CTX-M_, *bla*_SHV_, *bla*_IMP_, *bla*_NDM-1_	*sul3*	*cmlA1*	*tetM*, *tetW*	vanA	negative	*acrF*	*intl1-V*
ESBL*-producing K. pneumoniae* PX251755	0.90 10/11R	*bla*_SHV_, *bla*_KPC_, *bla*_IMP_, *bla*_NDM-1_, *bla*_TEM-1_	*sul2*	*cmlA1*	*tetA*, *tetB*, *tetM*	vanA	negative	negative	negative
ESBL*-producing K. pneumoniae* PX251756	0.81 9/11R	*bla*_SHV_*, bla*_KPC_, *bla*_OXA-48_, *bla*_OXA-1_	*sul1*, *sul3*	*cmx(A)*	*tetW*	vanA	negative	*acrF. adeA*	*intl-1*, *intl1-V*
ESBL*-producing K. pneumoniae* PX251757	0.72 8/11R	*bla*_CTX-M_, *bla*_SHV_, *bla*_IMP_, *bla*_NDM-1_, *bla*_TEM-1_	*sul3*	*cmx(A)*	*tetA*, *tetB*, *tetM*	vanA	negative	negative	*intl-1*
ESBL*-producing K. pneumoniae* PX251758	0.90 10/11R	*bla*_CTX-M_, *bla*_KPC_, *bla*_IMP_, *bla*_OXA-48_	*sul3*	*cmlA1*	*tetA*, *tetW*	vanA	negative	negative	*intl1-V*
ESBL*-producing K. pneumoniae* PX251759	0.90 10/11R	*bla*_SHV_, *bla*_*OXA-48*_, *bla*_OXA-1_, *bla*_TEM-1_	*sul1*, *sul2*	negative	*tetA*, *tetB*	vanA	negative	negative	negative
ESBL*-producing K. pneumoniae* PX251760	0.90 10/11R	*bla*_CTX-M_, *bla*_SHV_, *bla*_KPC_, *bla*_IMP_	*sul3*	*cmlA1*, *cmx(A)*	*tetA*, *tetB*, *tetW*	vanA	negative	*adeA*	*intl-1*
ESBL*-producing K. pneumoniae* PX251761	0.90 10/11R	*bla*_CTX-M_, *bla*_SHV_, *bla*_OXA-1_, *bla*_NDM-1_, *bla*_TEM-1_	*sul1*, *sul3*	*cmlA1*	*tetA*, *tetM*	vanA	negative	negative	negative
ESBL*-producing K. pneumoniae* PX251762	0.81 9/11R	*bla*_SHV_, *bla-*_*KPC*_, *bla*_IMP_, *bla*_OXA-48_, *bla*_NDM-1_, *bla*_*TEM-1*_	*sul2*, *sul3*	*cmx(A)*	*tetA*, *tetM*, *tetW*	vanA	negative	negative	*intl1-V*
ESBL*-producing E. coli* PX251776	0.90 10/11R	*bla*_CTX-M_, *bla*_SHV_, *bla*_OXA-1_, *bla*_NDM-1_	*sul2*, *sul3*	*cmlA1*	*tetA*, *tetM*	vanA	negative	*acrB*, *acrF*	*intl1-V*
ESBL*-producing E. coli* PX251767	0.90 10/11R	*bla*_SHV_, *bla*_KPC_, *bla*_IMP_, *bla*_OXA-1_, *bla*_NDM-1_	*sul1*, *sul2*	*cmlA1*, *cmx(A)*	*tetB*	vanA	negative	*acrA*, *acrF*, *adeA*	*intl-1*
ESBL*-producing E. coli* PX251768	0.90 10/11R	*bla*_CTX-M_, *bla*_SHV_, *bla*_KPC_, *bla*_IMP_, *bla*_OXA-48_, *bla*_OXA-1_, *bla*_NDM-1_, *bla*_TEM-1_	*sul2*, *sul3*	*cmlA1*, *cmx(A)*	*tetA*, *tetM*	vanA	negative	*acrB*, *acrF*, *acrR*	*intl-1*
*ESBL-producing E. coli* PX251769	0.81 9/11R	*bla*_CTX-M_, *bla*_SHV_, *bla*_IMP_, *bla*_NDM-1_	*sul1*, *sul3*	*cmx(A)*	*tetA*, *tetB*, *tetW*	vanA	negative	*acrA*, *acrF*, *adeA*	*intl-1*, *intl1-V*
ESBL*-producing E. coli* PX251771	0.90 10/11R	*bla*_SHV_, *bla*_OXA-48_, *bla*_OXA-1_, *bla*_NDM-1_, *bla*_TEM-1_	*sul1*, *sul2*, *sul3*	*cmlA1*	*tetA*, *tetB*, *tetW*	vanA	negative	*acrA*, *acrB*, *acrF*	negative
ESBL*-producing E. coli* PX251772	0.90 10/11R	*bla*_CTX-M_, *bla*_KPC_, *bla*_IMP_, *bla*_OXA-48_	*sul2*	*cmlA1*, *cmx(A)*	*tetA*, *tetM*	vanA	negative	*acrA*, *acrF*, *adeA*	*intl-1*, *intl1-V*
ESBL*-producing E. coli* PX251773	0.72 8/11R	*bla*_CTX-M_, *bla*_IMP_, *bla*_OXA-1_, *bla*_NDM-1_, *bla*_TEM-1_	*sul2*, *sul3*	*cmlA1*, *cmx(A)*	*tetA*, *tetM*, *tetW*	vanA	negative	*acrR*, *adeA*	*intl1-V*
ESBL*-producing E. coli* PX251774	0.90 10/11R	*bla*_SHV_, *bla*_KPC_, *bla*_OXA-48_, *bla*_OXA-1_, *bla*_NDM-1_	*sul1*	*cmlA1*	*tetA*, *tetM*	vanA	negative	*acrA*, *acrB*, *acrF*, *adeA*	*intl-1*
ESBL*-producing E. coli* PX251775	0.72 8/11R	*bla*_CTX-M_, *bla*_SHV_, *bla*_KPC_, *bla*_IMP_, *bla*_OXA-48_, *bla*_OXA-1_, *bla*_TEM-1_	*sul1*, *sul3*	*cmlA1*	*tetA*, *tetB*, *tetW*	vanA	negative	*acrA*, *acrF*, *acrR*	*intl-1*

b Results of phenotypic resistance profiling of species expressed as multiple antibiotic resistance index (MAR index).

The ARG profile of 67 Enterobacteriaceae and *Vibrio* spp. strains, isolated from RW (*n* = 20) and APSs (*n* = 47), covering 34 genes from major antibiotic classes, including CAM, sulfonamides, TET, β-lactams, glycopeptides, aminoglycosides, efflux pump genes, and class 1 integrons, is presented in Tables 3 and 4 respectively.

β-lactamase resistance included *bla*_CTX-M_, *bla*_CTX-M-15_, *bla*_SHV_, *bla*_KPC_, *bla*_IMP_, *bla*_VIM_, *bla*_OXA-48_, bla_OXA-1_, *bla*_NDM-1_, *bla*_TEM-1_, and *bla*_TEM-2_ genes. APSs strains exhibited distinct genotypic profiles of β-lactamase and carbapenem resistance genes. In ESBL-producing APSs-*E. coli* strains harbored *bla*_NDM-1_, *bla*_OXA-1_ and *bla*_SHV_ (77% each), *bla*_OXA-48_ (55%), and *bla*_TEM-1_ (44%). In ESBL-producing APSs-*K. pneumoniae* strains, *bla*_SHV_ was detected in 84%, and *bla*_TEM-1_, *bla*_NDM-1_, and *bla*_OXA-1_ in 46% of strains. These genes confer resistance to various β-lactam classes, including third-generation cephalosporins and penicillins. Additionally, carbapenemase genes, *bla*_KPC_ and *bla*_OXA-48_, were prevalent in these two strains, with *bla*_KPC_ at 55% and 69%, and *bla*_OXA-48_ at 55% and 53%, respectively, indicating extensive resistance to carbapenems and broad-spectrum β-lactams. Moreover, the ESBL-producing APSs-*K. oxytoca* strains showed a prevalence of *bla*_NDM-1_, *bla*_OXA-1_, *bla*_IMP_, *bla*_*KPC*_ (100%), *bla*_CTX-M-15_, *bla*_SHV_, and *bla*_CTX-M_ (66%), and *bla*_OXA-48_ (33%), reflecting resistance to a broad range of β-lactam antibiotics. Among APS *Vibrio* strains, *V. vulnificus* exhibited a prevalence of the *bla*_TEM-1_ gene (80%), *bla*_SHV_ (60%), and *bla*_CTX-M_ (40%). Additionally, *bla*_CTX-M_ was also detected in 44% of *V. parahaemolyticus* and in 37% of *V. cholerae* strains. While the other tested β-lactamases and carbapenem resistance genes were absent in *Vibrio* isolates. In conclusion, the resistance profiles of *Vibrio* species for β-lactamases genes were generally lower than those observed for ESBL-producing Enterobacteriaceae.

Furthermore, RW strains exhibited discrete genotypic profiles for β-lactamase and carbapenem resistance genes. Among ESBL-producing RW-*E. coli* strains, *bla*_CTX-M_, *bla*_TEM-1_, *bla*_KPC_, and *bla*_IMP_ were detected only in 50% of isolates. While, in ESBL-producing RW-*K. pneumoniae*, *bla*_SHV_ was present in 100% of isolates and *bla*_TEM-1_ in 50%. Notably, carbapenemase prevalence in RW was lower than in APS strains, with *bla*_OXA-48_ and *bla*_KPC_ detected in 25% and 50% of isolates, respectively. In RW-*V. parahaemolyticus and* RW*-V. cholerae*, only *bla*_SHV_ (40% and 33%, respectively) and *bla*_TEM-1_ (60% and 33%, respectively) were detected (Figures 3A and 3B).

**Figure 3:**
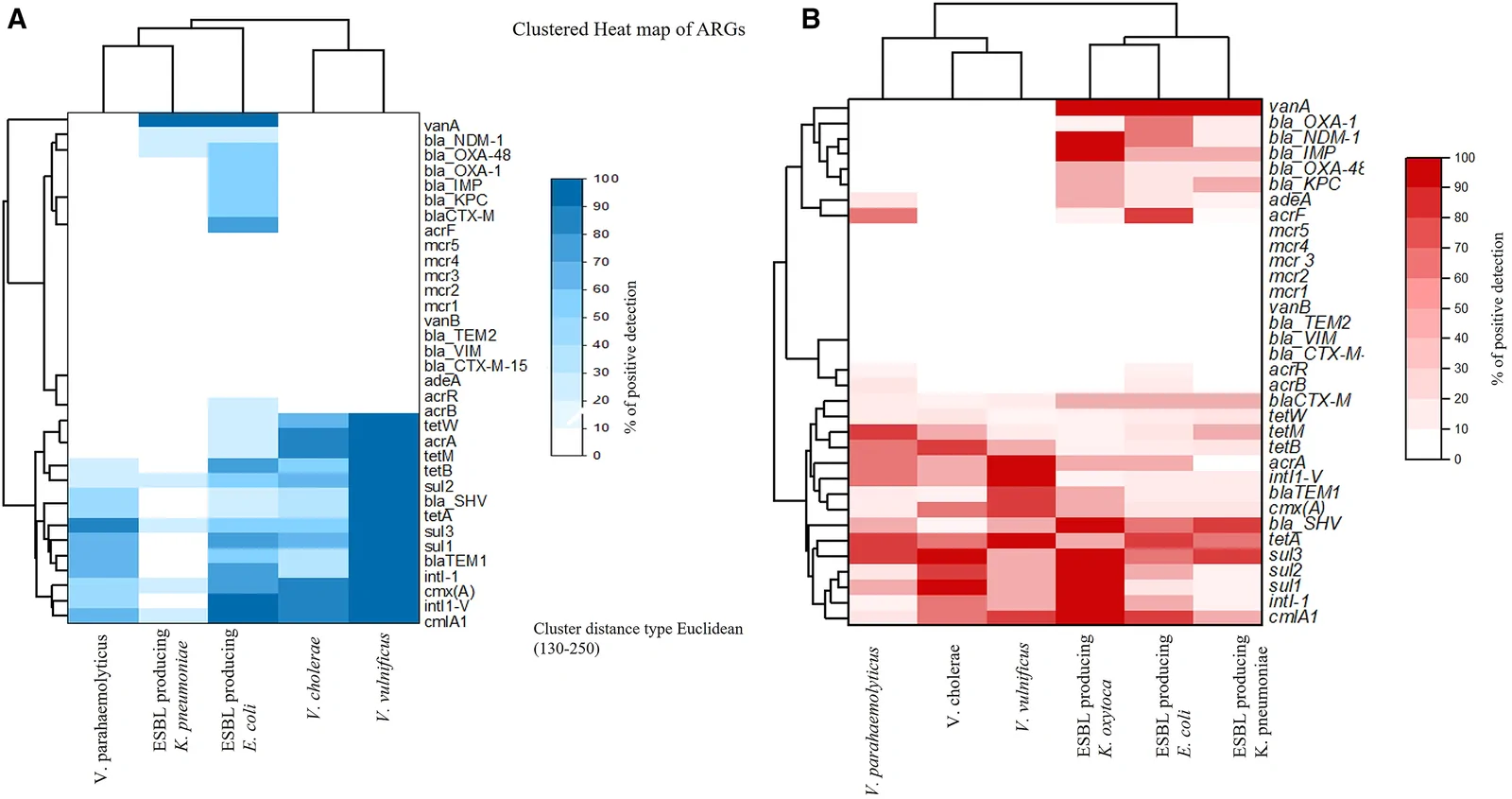
Genotypic resistance profile of isolated ARBs The heatmap analysis delineates the genotypic landscape of antimicrobial resistance genes across strains (A) RW and (B) APSs.

Resistance to CAM, *cmlA1*, and *cmx(A)* genes was detected in both APSs and RW strains, with notable variation in their frequencies among species. The *cmlA1* gene was frequently detected in ESBL-producing Enterobacteriaceae, *K. pneumoniae APSs 61%*, *RW 25%*, *E. coli APSs 88%*, *RW 100%*, *and K. oxytoca* APSs 100%. Among *Vibrio* species, 80% of APSs *V. vulnificus*, and 75% of *APSs V. cholerae* harbored the *cmlA1* gene. While the percentages of positive detection of *cmx(A)* gene were *K. oxytoca* 66%-APSs, *K. pneumoniae* 53%-APSs, 25%-RW, *E. coli* 55%-APSs, 75%-RW, *V. parahaemolyticus* 55%-APSs and 60%-RW, and *V. cholerae* 75%-APSs, 83%-RW (Figures 3A and 3B).

Resistance to sulfonamides, *sul1*, *sul2*, and *sul3* genes, *sul1* was prevalent in both APSs and RW strains, particularly in ESBL-producing *K. oxytoca* (APSs: 100%) and *V. cholerae* (APSs: 100%, RW: 66%). *sul2* was prevalent in *V. cholerae* (APSs: 87%, RW: 50%), *V. parahaemolyticus* (APSs: 55%, RW: 80%), ESBL-producing *K. oxytoca* (APSs: 100%), and ESBL-producing *E. coli* (APSs: 66%, RW: 50%). Similarly, *sul3* resistance was prevalent in *V. parahaemolyticus* and *V. cholerae*, reaching frequencies of 88% and 100% in APSs samples. In contrast, ESBL-producing *K. pneumoniae* showed lower prevalence rates for sulfonamide resistance, particularly for *sul1* (APSs: 38%, RW: 0%), *sul2* (APSs: 38%, RW: 25%), and *sul3* (APSs: 84%, RW: 25%) (Figures 3A and 3B).

TET resistance genes, *tetA*, *tetB*, *tetM*, and *tetW*, were highly diverse and positively detected across bacterial strains, with *tetA* and *tetM* being the most prevalent, as 75%–100% of APS strains of *V. vulnificus*, *V. parahaemolyticus*, and ESBL-producing *E. coli* harbored these two genes. Genes *tetA* and *tetM* were detected in ESBL-producing *K. pneumoniae* (APSs: 66% and 33%, respectively) and *K. oxytoca* (61% and 76%). In contrast, all four TET genes were absent in R.W ESBL-producing *K. pneumoniae*. Additionally, 75% of RW ESBL-producing *E. coli* isolates contained the *tetB* gene, whereas APS-associated strains exhibited a higher prevalence of *tetA* (88%) and *tetM* (55%), while *tetB* and *tetW* were found in only 44% of strains (Figures 3A and 3B).

Vancomycin resistance genes included *vanA* and *vanB*; the *vanA* gene was found in all APS and RW isolates of ESBL-producing *E. coli*, *K. pneumoniae* and *K. oxytoca*, but was absent in *Vibrio* strains.

Colistin resistance, including genes *mcr1*, *mcr2*, *mcr3*, *mcr4*, and *mcr5*, was absent in all strains.

The AcrAB efflux pump system (acrA, acrB, acrF, acrR, and adeA), a key player in multidrug resistance among Gram-negative bacteria, was also detected in our strains. Among ESBL-producing *E. coli*, *acrA* (APSs: 66%, RW:25%), *acrB* (APSs: 44%, RW: 25%), *adeA* (APSs: 55%, RW: 0%) and *acrF* (APSs: 88%, RW: 75%) were found. In APS ESBL-producing *K. pneumoniae*, only *acrF* (15%) and *adeA* (30%) were positively detected, while in RW ESBL-producing *K. pneumoniae*, all five genes were absent. Across *Vibrio* spp., the *acrA* gene was present in 100% of APSs-*V. vulnificus* and 77% of APSs-*V. parahaemolyticus*. While the rest of the efflux pump genes (*acrB*, *acrF*, *acrR*, and *adeA*) were absent in both RW and APSs *V. vulnificus* and *V. cholerae*, but in *V. parahaemolyticus*, these genes were positively detected (Figures 3A and 3B).

MGEs, *intI-1* and *intI1-V*, which encode integrase I, a key marker of integron-associated gene cassette mobilization, were widespread across all strains. The two genes were prevalent in ESBL-producing *E. coli* (75% and 100%, respectively) isolated from RW, compared with those isolated from APSs (66% and 44%, respectively). In contrast, ESBL-producing *K. pneumoniae* (38% and 46%, respectively) and ESBL-producing *K. oxytoca* (100% and 33%, respectively) harbored these two genes exclusively in APS samples. For *Vibrio* species, both genes, *intI-1* and *intI1-V*, were detected in APS and RW strains. RW *V. parahaemolyticus* (60% and 40%, respectively) and RW *V. cholerae* (33% and 83%, respectively), while for APSs *V. parahaemolyticus* (33% and 77%, respectively), APSs *V. vulnificus* (60% and 100%), and APSs *V. cholerae* (75% and 62%, respectively) (Figures 3A and 3B).

Hierarchical clustering analysis was employed to resolve both strain-related and gene-centric patterns (Figures 3A and 3B). Notably, among APS strains, *V. parahaemolyticus*, *V. vulnificus*, and *V. cholerae* formed a cohesive cluster, characterized by overlapping efflux pump-associated genes (*acrA*, *acrB*, and *acrR*) and TET resistance determinants (*tetA* and *tetB*), suggesting conserved strategies for multidrug resistance within this genus. In contrast, APS-associated ESBL-producing *K. pneumoniae*, *K. oxytoca*, and *E. coli* grouped into a divergent cluster enriched with extended-spectrum β-lactamase genes (*bla*_CTX-M-15_, *bla*_SHV_, and *bla*_TEM-1_) and carbapenem markers (*bla*_KPC_, *bla*_OXA-48_). RW *Vibrio* isolates were grouped into two distinct clusters based on their genotypic resistance profiles. Notably, RW-*V. parahaemolyticus* grouped with ESBL-producing Enterobacteriaceae, while RW-*V. vulnificus* and RW-*V. cholerae* were in separate groups. Gene co-occurrence networks further highlighted synergistic resistance mechanisms, including the linkage of sulfonamide resistance genes (*sul1* and *sul3*) with class 1 integrons, specifically *intI1-V*, suggesting the potential for horizontal transfer of MGEs. Additionally, the *tetM* and *tetW* genes clustered independently, indicating species-specific retention of TET resistance.

### Virulence genes in Vibrio strains

The *V. cholerae* strains isolated from APS and RW displayed a heterogeneous virulence gene profile, with positive results for *rtxC*, *ctxA*, and *hyla*. However, key cholera-associated genes, including *rtxA*, *ctxB*, *toxR*, *ompU*, and *vpi*, were negative. All *V. parahaemolyticus* isolates tested negative for the selected virulence genes *tdh*, *tlh*, *vppC*, *vgrG*, and *hcp*. The results for *V. vulnificus* showed that both virulence markers *vcgC* and *vcgE* were present in all strains. The prevalence of virulence determinants did not differ significantly between strains obtained from RW and APSs.

### Analysis of 16S rRNA sequences

Phylogenetic trees of 67 strains derived from APS and RW isolates of the Arrone River were generated using the neighbor-joining method and were created separately for ESBL-producing Enterobacteriaceae and *Vibrio* species, along with their respective reference strains, revealing distinct evolutionary relationships among the haplotypes (Figures 4A and 4B).

**Figure 4:**
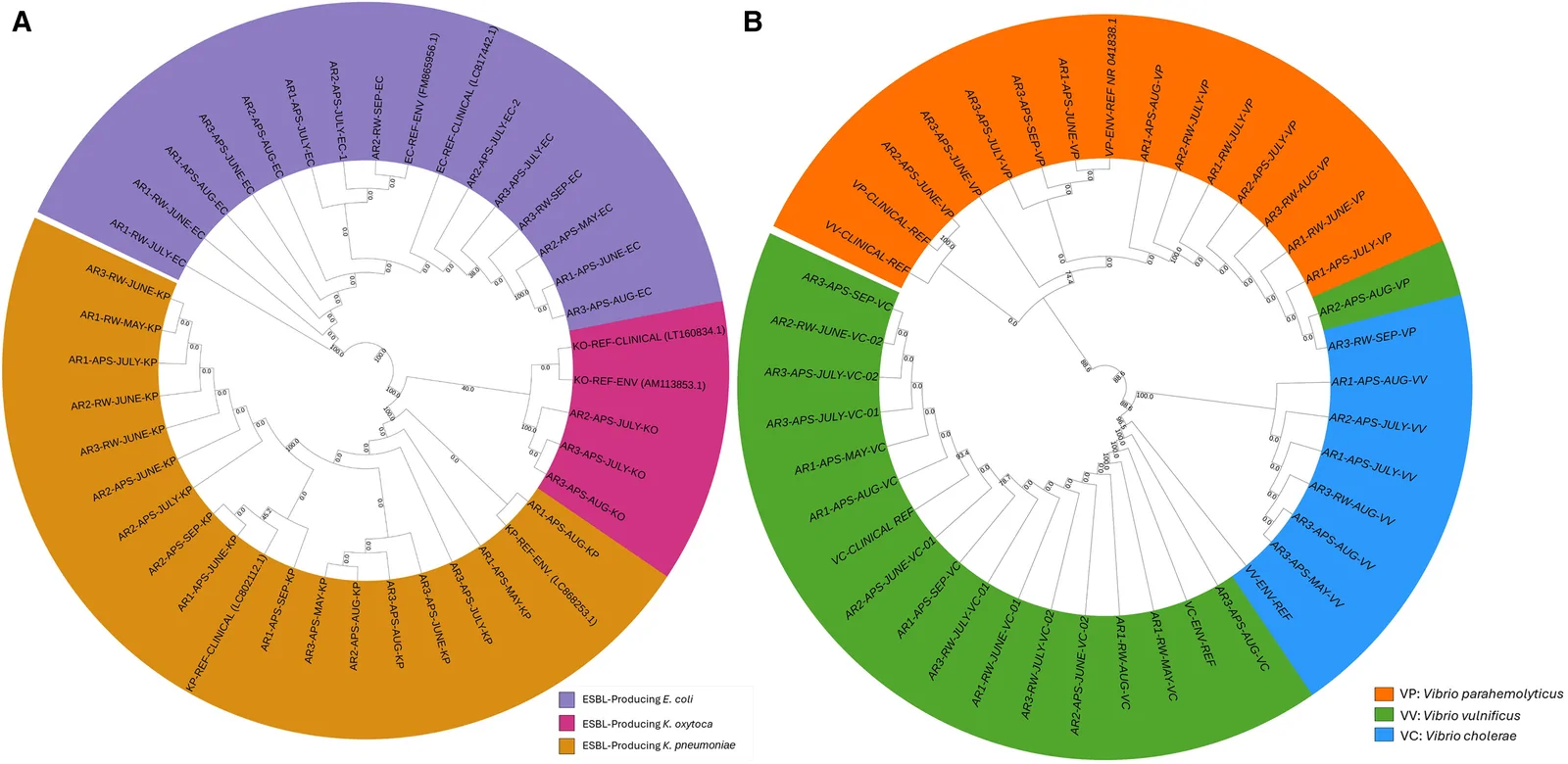
Phylogenetic tree of isolated ARBs (A) Phylogenetic tree aligned with reference strains: one clinical (in orange) and one environmental (in green) of the ESBL-producing *K. oxytoca*, *K. pneumoniae*, and *E. coli* sequences. (B) Phylogenetic tree of the *V. vulnificus*, *V. parahemolyticus*, and *V. cholerae*.

Figure 4A shows the phylogenetic tree of ESBL-producing *K. oxytoca*, *K. pneumoniae*, and *E. coli* sequences, aligned with known clinical reference strains (in orange) and environmental reference strains (in green). The phylogenetic tree of ESBL-producing Enterobacteriaceae showed two main clusters, for *E. coli* and *Klebsiella* spp. Within the *E. coli* cluster, there is a subcluster (AR2-APS-JULY-EC2) that forms a clade with the *E. coli* clinical reference strain (orange). The *Klebsiella* cluster comprises two subclusters: one for *K. oxytoca* and the other for *K. pneumoniae*. For *K. pneumoniae*, strain AR1-APS-SEP-KP was closely related to reference clinical strains, forming a distinct clade together with them. The environmental and clinical reference strains were correctly assigned to their respective species groups, confirming the correct taxonomic identities of strains.

The phylogenetic tree of *Vibrio* species (Figure 4B) shows three main clusters, corresponding to *V. vulnificus*, *V. parahaemolyticus*, and *V. cholerae*. Within the *V. parahaemolyticus* cluster, two strains (AR2-APS-JUNE-VP and AR2-APS-JUNE-VP-01) formed a distinct clade with clinical reference strains (orange). Similarly, in the *V. cholerae* cluster, strains were placed near the clinical reference strain. In contrast, the *V. vulnificus* strains obtained in this study were more closely associated with environmental reference strains, reflecting their environmental origin and distinct genetic profile.

These results highlight genetic differentiation among the studied species, confirming the reliability of their classification and providing insights into genetic diversity within clinical and environmental isolates of both Enterobacteriaceae and *Vibrio* species.

The obtained sequences of both RW and APS strains were submitted to NCBI gene bank under following accession number: *V. parahaemolyticus*: RW: PX251262 to PX251266; APSs: PX251267 to PX251275; *V. cholerae* RW: PX251247 to PX251252; APSs: PX251253 to PX251259 and PV785470; *V. vulnificus* RW: PX251241; APSs: PX251242 to PX251246; ESBL producing *K. pneumoniae* RW: PX251746 to PX251749; APSs: PX251750 to PX251762; ESBL producing *K. oxytoca*: APSs: PX251743 to PX251745; and ESBL producing *E. coli* RW: PX251763 to PX251766; APSs: PX251767 to PX251776.

## Discussion

The spread of ARBs and ARGs from aquatic ecosystems to humans poses a significant public health risk; however, their monitoring is not mandatory under the Water Framework Directive 2000/60/EC (WFD, 2000/60/EC) and the Marine Strategy Framework Directive (MSFD, 2008/56/EC)^20^^,^^21^

To improve understanding of the transmission pathways of ARBs, our work focused on detecting their proliferation in aquatic environments, particularly in transitional waters such as estuaries and coastal zones, where they pose immediate risks to human, animal, and environmental health. Transitional waters are especially vulnerable because they act as mixing zones between freshwater and seawater, facilitating not only the persistence and dissemination of ARBs but also the accumulation of plastic waste.^22^ However, pathogens in aquatic ecosystems are present at low levels, so the sampling method is a crucial factor in studying their prevalence and dissemination.^16^ Hence, a key challenge in our study was to develop a sampling tool to detect ARBs and ARGs at three sites along a river continuum, from the source to the river mouth, without abstracting a large volume of water. To address this, we developed a sampling tool based on APSs made from plastic polymers representative of the most common plastic contaminants in aquatic environments. The APSs comprised seven polymer types, each providing a suitable surface for bacterial attachment and colonization, thereby functioning as efficient bacterial traps (Figure 1 from A to D). Recent studies have demonstrated that plastic serves as a suitable substrate for bacterial proliferation and ARG transmission, forming the so-called plastisphere.^23^^,^^24^ The hydrophobic properties, zeta potentials, and surface charges of these polymers promote rapid bacterial adhesion and biofilm formation, thereby enhancing bacterial persistence and improving sampling efficiency and detection sensitivity.^25^

**Figure 1:**
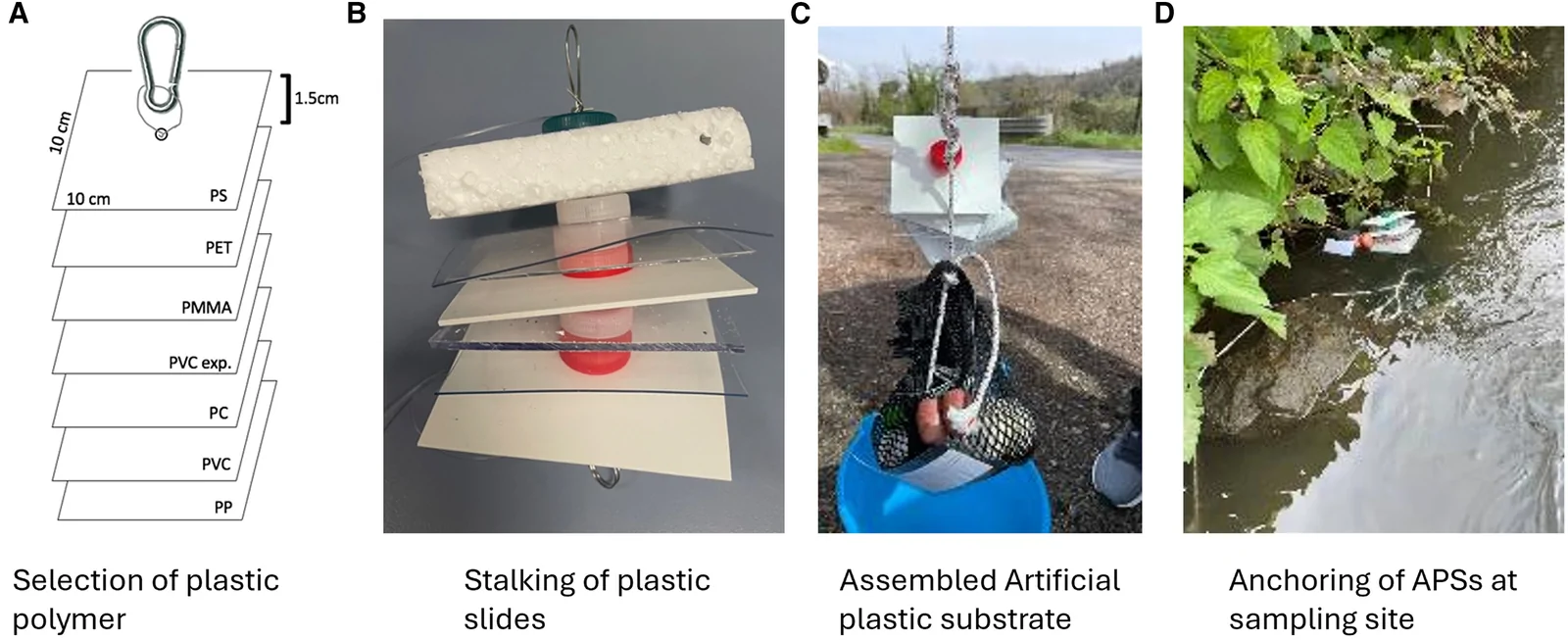
Structure of artificial plastic substrates (A) The seven plastic polymers. (B) APS and the spacer between polymers. (C) Net with stone for anchoring. (D) APS on site. Artificial plastic substrate (APSs) sampling tool developed using polystyrene (PS), polyethene terephthalate (PET), polymethyl methacrylate (PMMA), expanded polyvinyl chloride (PVC expanded), polycarbonate (PC), polyvinyl chloride (PVC), and polypropylene (PP).

Five monthly samples were conducted at the three study sites, and both RW and APSs samples were analyzed. We aimed to deepen understanding of the role of aquatic ecosystems in the spread of AMR within a One Health perspective, focusing not only on the monitoring of a single indicator, ESBL-producing *E. coli* (WHO, 2021), but also on ESBL-producing *Klebsiella* spp. and *Vibrio* spp. In fact, 127 targeted bacteria were recovered and identified, including ESBL-producing Enterobacteriaceae and *Vibrio* spp. The recurrent detection of ESBL-producing *Klebsiella pneumoniae*, *K. oxytoca*, and *E. coli*, together with *Vibrio* spp., confirms the role of surface waters as reservoirs of pathogenic bacteria (Figure 2B).

**Figure 2:**
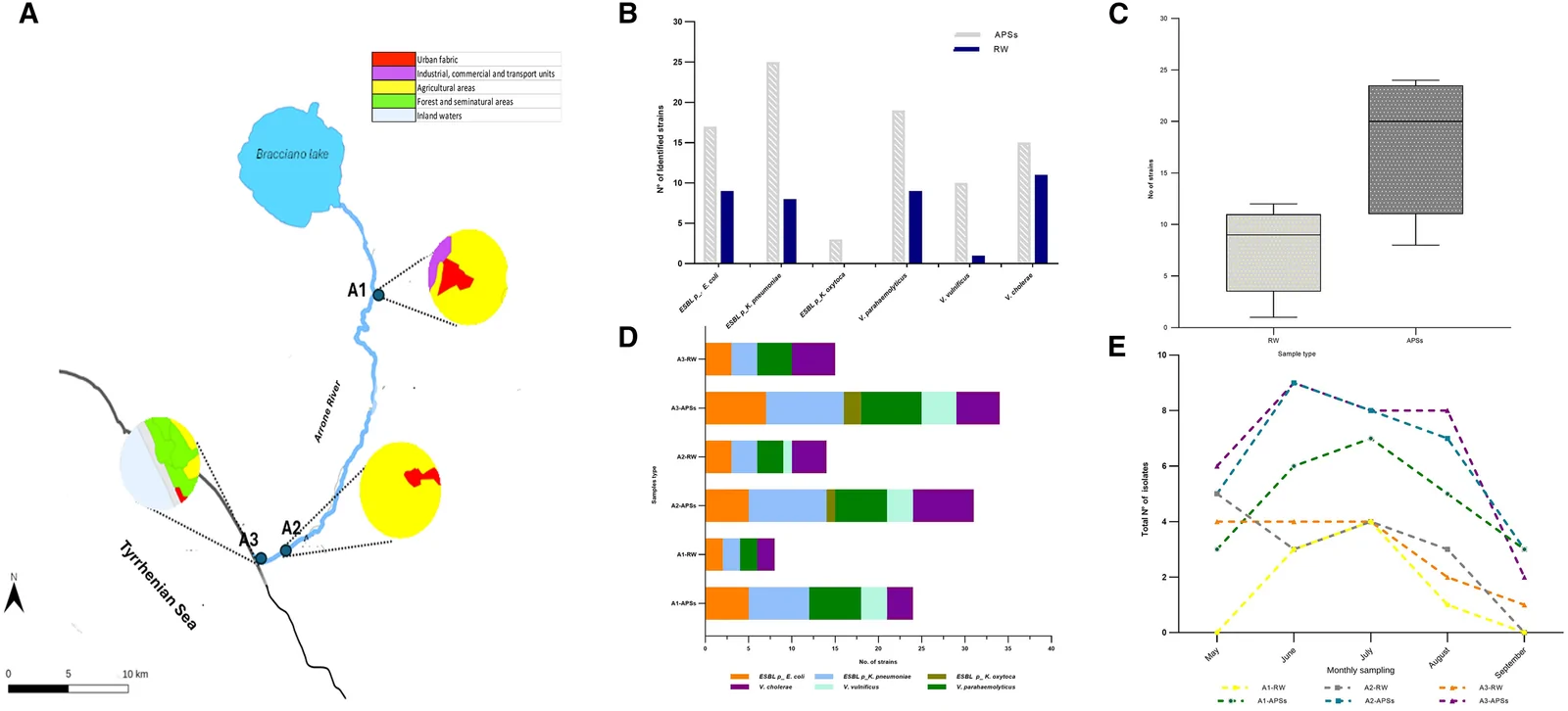
ARBs recovery from APSs and RW along the Arrone River: spatial and temporal distribution (A) Land use and geographical location of the three study sites along the Arrone River: A1, Osteria Nuova (N 42° 02′ 13″, E 12° 18′ 48″); A2, Castello Maccarese (N 41° 52′ 30″, E 12° 12′ 38″); A3, Fregene (N 41° 52′ 08″, E 12° 10′ 48″). Circular insets show land use within a 1 km radius of each site. Scale bars, 10 km on bottom left corner. (B) Number of ARBs detected from RW and APSs. (C) The boxplot reveals a diversity of ARBs isolated from APSs and the RW sample (t = 2.43, *p* ≤ 0.05). (D) Spatial variation in pathogen recovery among sites. while spatial variation among sites was significant only for APSs (*p* = 0.032) but not for RW samples (*p* = 0.15). (E) Temporal variations in pathogen recovery across sampling campaigns. Repeated-measures ANOVA also revealed significant temporal variation across months (*p* < 0.001).

Spatial variability observed along the river course highlights the influence of anthropogenic pressures, which extend from upstream areas to the river mouth and exert additional impacts on transitional and marine environments (Figure 2D). Moreover, the consistent detection of these pathogens across sampling campaigns indicates established environmental persistence rather than sporadic contamination, underscoring the need for integrated AMR surveillance and management strategies that encompass aquatic environments alongside clinical and agricultural sectors.

Notably, APSs showed a higher prevalence of target pathogenic bacteria than RW samples, further validating their effectiveness as a sensitive tool for detecting ARBs in aquatic ecosystems without requiring large-volume water sampling. Hence, our methodology provides a reliable and efficient means of assessing aquatic ecosystems as reservoirs of AMR, particularly given the widespread presence of floating plastic debris, which may represent a novel exposure pathway from a One Health perspective.

Temporal analysis revealed higher pathogen prevalence during June, July, and August, coinciding with elevated water temperatures and peak tourism and recreational activities, while lower prevalence was observed in May and September, corresponding to the beginning and end of the bathing season.

Our findings also suggest a potential link between seasonal human activity, environmental conditions, and microbial proliferation in aquatic systems. Temperature, a significant environmental factor, influences their planktonic and biofilm forms, with warmer sea-surface temperatures promoting their growth and increasing infection rates, particularly in summer.^26^^,^^27^ Not least, climate stressors such as temperature and salinity can affect the survival and spread of pathogens, for example, exacerbating vibriosis by prolonging warm sea-surface temperatures.^28^ The results also underscore the potential of APSs as a robust, low-cost, and field-adaptable tool for monitoring ARB dissemination in aquatic ecosystems, particularly in developing countries facing climate-driven fluctuations in terms of water quality and availability.

Moreover, antibiotic susceptibility testing revealed that a high proportion of the APS-associated isolates were resistant to all antibiotics tested. These findings not only elucidate the genetic landscape of AMR within APS strains but also underscore the substantial potential for interspecies dissemination of resistance determinants, particularly among high-risk ESBL-producing pathogens.^29^ The near-ubiquitous resistance to WHO-priority antibiotics, including CIP,CAZ, and IMP, observed in our APS strains, poses serious public health concerns. Notably, the persistence of IMP resistance among ESBL-producing Enterobacteriaceae in the present study underscores the urgent need for enhanced surveillance of carbapenem resistance genes in aquatic environments.

*Vibrio* spp. also exhibited substantial resistance to clinically essential antibiotic classes, including fluoroquinolones, β-lactamases, aminoglycosides, and TET. This is particularly concerning given their role as foodborne and waterborne pathogens with significant zoonotic and human health implications within the One Health framework.^30^ The higher resistance observed in *Vibrio* isolates from APS samples suggests a strong capacity to acquire and maintain resistance under sustained selective pressure. The detection of these ARBs on plastic further highlights the issue, as plastics may act as vectors, facilitating the spread of pan-resistant pathogens into drinking water systems, reclaimed and recycled water, and food chains.^31^

In our isolates, pan-resistance was accompanied by very high MAR index values (> 0.7). These patterns indicate exposure of strains to intense antibiotic selection pressures and contamination from high-risk anthropogenic sources rather than typical environmental backgrounds. These findings underscore the urgent need for targeted source-tracking efforts and mitigation strategies, particularly to address wastewater discharges and agricultural runoff.

Among the ARGs in ESBL-producing Enterobacteriaceae isolated from both sample types, β-lactamases (*bla*_*CTX-M*_, *bla*_*SHV*_, and *bla*_*TEM*_) were frequently detected. These genes facilitate their transfer not only within species but also across diverse bacterial taxa, often in association with additional resistance determinants that promote multidrug resistance. Furthermore, in APSs strains, *Klebsiella pneumoniae carbapenemases* (*bla*_*KPC*_), *New Delhi metallo-β-lactamase* (*bla*_*NDM*_), and *oxacillinase-48-type* (*bla*_*OXA-48*_) were also detected. Along with the detection of β-lactamase resistance genes, the positive detection of genes belonging to other antibiotic classes, including tetracyclines, aminoglycosides, sulfonamides, chloramphenicols, and efflux pump genes in both sample types, indicates a significant prevalence of antibiotic contamination, which may contribute to the selection and dissemination of resistant bacterial strains. Additionally, the high prevalence of class 1 integrons in APS and RW-associated strains further amplifies public health concerns. Integrons, MGEs, may enable the horizontal transfer of gene cassettes and therefore could play a pivotal role in the dissemination of AMR across environmental and clinical settings.^32^^,^^33^

In a nutshell, the coexistence of multidrug-resistant *Vibrio* species and ESBL-producing Enterobacterales in the Arrone River not only represents a combined pathogenic threat but also raises concerns about genetic exchange and co-selection of resistance traits in a shared aquatic niche.^34^^,^^35^

Furthermore, in the *V. cholerae* strains, the detection of virulence-associated genes such as *rtxC* and *ctxA*, which are involved in cholera toxin production, together with *hylA*, associated with hyaluronidase activity, suggests that these strains may have the capacity to damage host tissues and cause localized infections or gastroenteritis under favorable conditions, despite not being fully virulent.^30^ However, the absence of key virulence determinants, including *rtxA*, *ctxB*, *toxR*, *ompU*, and *vpi*, indicates that the obtained strains are likely attenuated or belong to non-epidemic strains, consistent with reduced virulence potential. As cholera toxin requires both *ctxA* and *ctxB* for functional toxin production, the detection of *ctxA* alone does not confirm toxigenic potential. Therefore, our findings indicate the presence of virulence-associated genes rather than evidence of functional toxin production or pathogenicity. Moreover, unlike the divergence observed for ARGs, no significant difference in the prevalence of virulence genes was found between RW and APS strains. This pattern suggests that virulence genes are broadly conserved across environmental niches and may not be preferentially enriched by plastisphere-associated selection pressures, consistent with their frequent chromosomal localization and host-contingent regulation.

The phylogenetic analysis showed a close relationship among the clinical reference strains, our APS, and RW strains. The results of this study advance the understanding of the role of aquatic ecosystems in the dissemination of AMR within a One Health framework, focusing on the Arrone River basin in Italy. Rather than limiting the analysis to a single indicator, ESBL-producing *E. coli* across the human, animal, and environmental sectors, we also investigated ESBL-producing *Klebsiella* spp. and *Vibrio* spp. Furthermore, we developed and implemented an innovative sampling approach using APSs composed of the leading polymers representative of environmental plastic waste. These APSs enabled the tracking of antimicrobial-resistant bacteria and the assessment of plastics as potential transmission vectors, thereby facilitating the spread of AMR from inland freshwater systems through transitional waters into the marine environment.

In a nutshell, the environmental fate of ARB and their AMR genes in aquatic ecosystems remains a critical One Health concern for protecting both human and animal health. Detecting pathogenic bacteria in aquatic ecosystems is a significant challenge, owing to the quality and quantity of sample collection, as pathogens are often present at trace levels. The APSs tool was optimized by increasing the number of polymers in the prototype, which better represents the most common plastic debris encountered in aquatic ecosystems and their sizes. The use of standardized size 10 × 10 cm polymer tiles provided a consistent colonization surface area (100 cm^2^ per polymer type), ensuring comparability across substrates while enhancing the effective area available for microbial attachment. Our findings demonstrate that the APSs tool is highly effective for detecting Multi drug resistant bacteria (MDR) bacteria in aquatic environments affected by extreme weather events, such as drought and water scarcity, without requiring large-volume water sampling. Importantly, APSs deployment offers a reproducible, practical, and scalable approach for the concurrent detection of both ARBs and ARGs.

The results also underscore the potential of APSs as a robust, low-cost, and field-adaptable tool for monitoring AMR dissemination in aquatic ecosystems, particularly in developing countries facing climate-driven fluctuations in terms of water quality and availability. This approach aligns with the One Health framework by linking environmental reservoirs of resistance to broader public health risks.

This study provides insights into the role of plastic as a vehicle for the dissemination of AMR. Furthermore, we provide scientific evidence not only on the monitoring of a single indicator, theESBL-producing *E. coli*, across the human, animal, and environmental sectors within a One Health approach, but also on ESBL *Klebsiella* and *Vibrio*, offering a broader picture of the role of aquatic ecosystems and transmission pathways of AMR genes. The detection of ARBs and ARGs on plastic substrates highlights the capacity of plastics to act not merely as passive carriers but as dynamic hotspots for bacterial colonization and horizontal gene transfer (HGT) events. The stable surface and high microbial density associated with these substrates likely promote plasmid-mediated exchange, thereby amplifying the persistence and spread of ESBL resistance and other pathogenic bacteria within the aquatic ecosystem. Moreover, the phylogenetic tree showed a close relationship among the clinical reference strains, our APS, and RW strains, reinforcing the urgent need for scientific evidence from the environmental sector, in line with the One Health approach, to combat the antibiotic resistance pandemic. Our findings extend previous studies documenting the presence of ARGs in transitional waters. Our data suggest that plastic waste may constitute an emerging route of exposure to resistant pathogens, particularly among individuals engaged in recreational water activities.

Overall, this study highlights the intertwined challenges of AMR, plastic pollution, and climate change, emphasizing the need for integrated surveillance, policy interventions, and public awareness strategies. By providing an innovative, field-deployable monitoring tool, the APSs approach advances our understanding of the environmental dimensions of AMR. It supports efforts to mitigate its spread under the One Health paradigm. The deployment of APSs in developing countries offers a practical means of enhancing microbial detection in aquatic systems. In regions increasingly stressed by water scarcity and climate-driven fluctuations in water quality, APSs provide a low-cost, scalable alternative to conventional monitoring methods. Their early detection of pathogenic bacteria can improve water resource management and strengthen public health resilience in vulnerable settings.

### Limitations of the study

To date, APSs have mainly been used to detect targeted pathogenic bacteria in inland and transitional waters. Plastic-associated microbial communities are highly diverse and can include pathogenic bacteria, antibiotic-resistant microorganisms, viruses, fungi, and other biological contaminants. At the same time, plastics can also accumulate a wide range of chemical pollutants. Whether APSs can reliably detect such a broad spectrum of biological and chemical hazards, either individually or simultaneously, requires further investigation.

The complexity of the plastisphere may also represent an important challenge for APS-based detection. Biofilm formation, microbial interactions, plastic polymer type, degree of weathering, and environmental conditions such as temperature and salinity may affect the composition and abundance of microorganisms associated with plastic surfaces and, consequently, their detectability.

Future studies should therefore validate APSs across a wider range of environmental compartments, plastic types, geographic regions, and microbial communities. Such applications could provide valuable information on plastic particles as potential vectors for the environmental dissemination of pathogens.

Another limitation is the current emphasis on waterborne pathogens. Because plastics are highly mobile and can be transported between aquatic, terrestrial, and atmospheric environments, future research should assess whether APSs can be extended to surveillance of marine- and air-transmitted pathogens.

Addressing these limitations would support the transition of APSs from a targeted pathogen-detection method toward a broader environmental surveillance tool.

## Resource availability

### Lead contact

Requests for further information and resources should be directed to and will be fulfilled by the lead contact, Stefania Marcheggiani (stefania.marcheggiani@iss.it).

### Materials availability

This study did not generate new unique reagents.

### Data and code availability

• The original 16S rRNA sequences dataset generated in this paper have been deposited on the NCBI Genome Bank (publicly available at https://www.ncbi.nlm.nih.gov/gene/) with the accession number mentioned in the key resources table.
• Any additional information in this paper is available from the lead contact upon request.
• This paper does not generate any original code.

## Acknowledgments

This research has received funding from the HORIZON-RIA - HORIZON Research and Innovation Actions, EU Project BlueAdapt, under grant agreement no. 101057764.

## Author contributions

S.M., writing – original draft, writing – review and editing, validation, methodology, investigation, formal analysis, funding acquisition, data curation, and conceptualization; I.F., writing – original draft, formal analysis, and data curation; M.R.L., visualization and software; F.C., support for sampling; M.M., M.B.N., E.B., L.M., S.W., and K.P., review and editing.

## Declaration of interests

Authors have no conflicts of interest to declare.

## STAR★Methods

### Key resources table

**Table:** 

REAGENT or RESOURCE	SOURCE	IDENTIFIER
**Chemicals, peptides, and recombinant proteins**		

Mueller-Hinton (MH) broth	Condalab, Madrid, Spain	Lot: 510302
ESBL chromogenic agar	Condalab, Madrid, Spain	Lot: 603055
ESBL chromogenic agar supplement	Condalab, Madrid, Spain	Lot: 165562
Vibrio chromogenic agar	Condalab, Madrid, Spain	Lot: 507033
Mueller–Hinton (MH) agar	Condalab, Madrid, Spain	Lot: 511191
Cryoinstant beads	VWR Chemicals, Milano, Italy.	Lot: 147302
API-20E	BioMeriuex, Grassina, Italy	Lot: 1010795010
API-20NE	BioMeriuex, Grassina, Italy	Lot: 1010924420
DNA Extraction kit	GRISP, Porto, Portugal	Lot: 7EA0408F
PCR and Gel band Purification kit	GRISP, Porto, Portugal	Lot:7E81021A
Sanger DNA sequencing	Microsynth, Göttingen, Germany	https://www.microsynth.com/sanger_sequencing_en.html
Gentamicin	Condalab, Madrid, Spain	Lot: C27710A
Kanamycin	Condalab, Madrid, Spain	Lot: C01301b
Ciprofloxacin	Condalab, Madrid, Spain	Lot: 210325A
Ceftazidime	Condalab, Madrid, Spain	Lot: 210604B
Cefoxitin	Condalab, Madrid, Spain	Lot: C10903B
Imipenem	Liofilchem. Teramo, Italy	Lot: 102623005
Erythromycin	Condalab, Madrid, Spain	Lot: C07102B
Nalidixic Acid	Condalab, Madrid, Spain	Lot:72622
Tetracycline	Condalab, Madrid, Spain	Lot: C06602B
Chloramphenicol	Condalab, Madrid, Spain	Lot: 210610J
Sulfamethoxazole	Condalab, Madrid, Spain	Lot: C11909A
TBE	GRISP, Porto, Portugal	Lot: 7E31102B
100 bp ladder	SMOBIO, Hsinchu County, Taiwan	Lot: DM23002110122_1
Taq DNA polymerase	GRISP, Porto, Portugal	Lot: 7E50506B
GRS Taq polymerase buffer	GRISP, Porto, Portugal	Lot:7E50506B
dNTPs	GRISP, Porto, Portugal	Lot: GP011.0411
1X Gel Red	Biotium, Fremont, USA	Lot: 21G0922

**Deposited data**		

VP1-AR1-RW-JUNE	This Study	*Vibrio parahemolyticus* GenBank: PX251262
VP2-AR1-RW-JULY	This Study	*Vibrio parahemolyticus* GenBank: PX251263
VP3-AR2-RW-JULY	This Study	*Vibrio parahemolyticus* GenBank: PX251264
VP4-AR3-RW-AUGUST	This Study	*Vibrio parahemolyticus* GenBank: PX251265
VP5-AR3-RW-SEPTEMBER	This Study	*Vibrio parahemolyticus* GenBank: PX251266
VC1-AR1-RW-MAY	This Study	*Vibrio cholerae* GenBank: PX251247
VC2-AR1- RW -JUNE	This Study	*Vibrio cholerae* GenBank: PX251248
VC3-AR2- RW -JUNE	This Study	*Vibrio cholerae* GenBank: PX251249
VC4-AR3- RW -JULY	This Study	*Vibrio cholerae* GenBank: PX251250
VC5-AR3-RW-JULY	This Study	*Vibrio cholerae* GenBank: PX251251
VC6-AR1-RW-AUGUST	This Study	*Vibrio cholerae* GenBank: PX251252
VV1-AR3-RW-JULY	This Study	*Vibrio vulnificus* GenBank: PX251241
KP1-AR1-RW-MAY	This Study	ESBL-producing *Klebsiella pneumoniae* GenBank: PX251746
KP2-AR2-RW-JUNE	This Study	ESBL-producing *Klebsiella pneumoniae* GenBank: PX251747
KP3-AR3-RW-JUNE	This Study	ESBL-producing *Klebsiella pneumoniae* GenBank: PX251748
KP4-AR3-RW-JUNE	This Study	ESBL-producing *Klebsiella pneumoniae* GenBank: PX251749
EC1-AR1-RW-JUNE	This Study	ESBL-producing *Escherichia coli* GenBank: PX251763
EC2-AR1-RW-JULY	This Study	ESBL-producing *Escherichia coli* GenBank: PX251764
EC3-AR2-RW-SEPTEMBER	This Study	ESBL-producing *Escherichia coli* GenBank: PX251765
EC4-AR3-RW-SEPTEMBER	This Study	ESBL-producing *Escherichia coli* GenBank: PX251766
VP1-AR1-APS-JUNE	This Study	*Vibrio parahemolyticus* GenBank: PX251267
VP2-AR1-APS-JULY	This Study	*Vibrio parahemolyticus* GenBank: PX251268
VP3-AR1-APS-AUGUST	This Study	*Vibrio parahemolyticus* GenBank: PX251269
VP4-AR2-APS-JUNE	This Study	*Vibrio parahemolyticus* GenBank: PX251270
VP5-AR2-APS-JULY	This Study	*Vibrio parahemolyticus* GenBank: PX251271
VP6-AR2-APS-AUGUST	This Study	*Vibrio parahemolyticus* GenBank: PX251272
VP7-AR3-APS-JUNE	This Study	*Vibrio parahemolyticus* GenBank: PX251273
VP8-AR3-APS-JULY	This Study	*Vibrio parahemolyticus* GenBank: PX251274
VP9-AR3-APS-SEPTEMBER	This Study	*Vibrio parahemolyticus* GenBank: PX251275
VC1-AR1-APS-JUNE	This Study	*Vibrio cholerae* GenBank: PX251259
VC2-AR1-APS-JULY	This Study	*Vibrio cholerae* GenBank: PV785470
VC3-AR1-APS-AUGUST	This Study	*Vibrio cholerae* GenBank: PX251253
VC4-AR2-APS-MAY	This Study	*Vibrio cholerae* GenBank: PX251254
VC5-AR2-APS-JUNE	This Study	*Vibrio cholerae* GenBank: PX251255
VC6-AR3-APS-MAY	This Study	*Vibrio cholerae* GenBank: PX251256
VC7-AR3-APS-JUNE	This Study	*Vibrio cholerae* GenBank: PX251257
VC8-AR3-APS-AUGUST	This Study	*Vibrio cholerae* GenBank: PX251258
VV1-AR1-APS-JULY	This Study	*Vibrio vulnificus* GenBank: PX251242
VV2-AR1-APS-AUGUST	This Study	*Vibrio vulnificus* GenBank: PX251243
VV3-AR2-APS-JULY	This Study	*Vibrio vulnificus* GenBank: PX251244
VV4-AR3-APS-MAY	This Study	*Vibrio vulnificus* GenBank: PX251245
VV5-AR3-APS-AUGUST	This Study	*Vibrio vulnificus* GenBank: PX251246
KO1-AR2-APS-JULY	This Study	ESBL-producing *Klebsiella oxytoca* GenBank: PX251743
KO2-AR3-APS-JULY	This Study	ESBL-producing *Klebsiella oxytoca* GenBank: PX251744
KO3-AR3-APS-AUGUST	This Study	ESBL-producing *Klebsiella oxytoca* GenBank: PX251745
KP1-AR1-APS-MAY	This Study	ESBL-producing *Klebsiella pneumoniae* GenBank: PX251750
KP2-AR1-APS-JUNE	This Study	ESBL-producing *Klebsiella pneumoniae* GenBank: PX251751
KP3-AR1-APS-JULY	This Study	ESBL-producing *Klebsiella pneumoniae* GenBank: PX251752
KP4-AR1-APS-AUGUST	This Study	ESBL-producing *Klebsiella pneumoniae* GenBank: PX251753
KP5-AR1-APS-SEPTEMBER	This Study	ESBL-producing *Klebsiella pneumoniae* GenBank: PX251754
KP6-AR2-APS-JUNE	This Study	ESBL-producing *Klebsiella pneumoniae* GenBank: PX251755
KP7-AR2-APS-JULY	This Study	ESBL-producing *Klebsiella pneumoniae* GenBank: PX251756
KP8-AR2-APS-AUGUST	This Study	ESBL-producing *Klebsiella pneumoniae* GenBank: PX251757
KP9-AR2-APS-SEPTEMBER	This Study	ESBL-producing *Klebsiella pneumoniae* GenBank: PX251758
KP10-AR3-APS-MAY	This Study	ESBL-producing *Klebsiella pneumoniae* GenBank: PX251759
KP11-AR3-APS-JUNE	This Study	ESBL-producing *Klebsiella pneumoniae* GenBank: PX251760
KP12-AR3-APS-JULY	This Study	ESBL-producing *Klebsiella pneumoniae* GenBank: PX251761
KP13-AR3-APS-AUGUST	This Study	ESBL-producing *Klebsiella pneumoniae* GenBank: PX251762
EC1-AR1-APS-JUNE	This Study	ESBL-producing *Escherichia coli* GenBank: PX251776
EC2-AR1-APS-JULY	This Study	ESBL-producing *Escherichia coli* GenBank: PX251767
EC3-AR1-APS-AUGUST	This Study	ESBL-producing *Escherichia coli* GenBank: PX251768
EC4-AR2-APS-MAY	This Study	ESBL-producing *Escherichia coli* GenBank: PX251769
EC5-AR2-APS-JULY	This Study	ESBL-producing *Escherichia coli* GenBank: PX251771
EC6-AR2-APS-AUGUST	This Study	ESBL-producing *Escherichia coli* GenBank: PX251772
EC7-AR3-APS-JUNE	This Study	ESBL-producing *Escherichia coli* GenBank: PX251773
EC8-AR3-APS-JULY	This Study	ESBL-producing *Escherichia coli* GenBank: PX251774
EC9-AR3-APS-AUGUST	This Study	ESBL-producing *Escherichia coli* GenBank: PX251775

**Oligonucleotides**		

intI1-V	Zhang et al.^36^	F: TCATGGCTTGTTATGACTGT R: GTAGGGCTTATTATGCACGC
intI-1	Zhang et al.^37^	F: GGCTTCGTGATGCCTGCTT R: CATTCCTGGCCGTGGTTCT
bla_CTX-M	Zhang et al.^37^	F: GGAGGCGTGACGGCTTTT R: TTCAGTGCGATCCAGACGAA
bla_SHV	Zhang et al.^37^	F: TCCCATGATGAGCACCTTTAAA R: TTCGTCACCGGCATCCA
bla_TEM	Zhang et al.^37^	F: AGCATCTTACGGATGGCATGA R: TCCTCCGATCGTTGTCAGAAGT
bla_OXA-1	Magiorakos et al.^1^	F: GGCACCAGATTCAACTTTCAAG R: GACCCCAAGTTTCCTGTAAGTG
bla_IMP	Magiorakos et al.^38^	F: CATTCAAGGGCTTTCTTGCTGC R: ACGACGGCATAGTCATTTGC
bla_VIM	Magiorakos et al.^38^	F: TTGACACTCCATTTACDG R: GATYGAGAATTAAGCCACYCT
bla_KPC	Magiorakos et al.^38^	F: GATGGTGTTTGGTCGCATA R: CGAATGCGCAGCACCAG
bla_OXA-48	Magiorakos et al.^38^	F: GCTTGATCGCCCTCGATT R: GATTTGCTCCGTGGCCGAAA
bla_NDM-1	Magiorakos et al.^38^	F: GGTTTGGCGATCTGGTTTTC R: CGGAATGGCTCATCACGATC
cmlA1	Zhang et al.^37^	F: TAGGAAGCATCGGAACGTTGAT R: CAGACCGAGCACGACTGTTG
cmx(A)	Zhang et al.^37^	F: GCGATCGCCATCCTCTGT R: TCGACACGGAGCCTTGGT
tetA	Zhang et al.^37^	F: GCTGTTTGTTCTGCCGGAAA R: GGTTAAGTTCCTTGAACGCAAACT
tetB	Zhang et al.^37^	F: AGTGCGCTTTGGATGCTGTA R: AGCCCCAGTAGCTCCTGTGA
tetM	Zhang et al.^37^	F: CATCATAGACACGCCAGGACATAT R: CGCCATCTTTTGCAGAAATCA
tetW	Zhang et al.^37^	F: ATGAACATTCCCACCGTTATCTTT R: ATATCGGCGGAGAGCTTATCC
sul1	Zhang et al.^37^	F: CGCACCGGAAACATCGCTGCAC R: TGAAGTTCCGCCGCAAGGCTCG
sul2	Zhang et al.^37^	F: TCCGGTGGAGGCCGGTATCTGG R: CGGGAATGCCATCTGCCTTGAG
sul3	Zhang et al.^37^	F: TCCGTTCAGCGAATTGGTGCAG R: TTCGTTCACGCCTTACACCAGC
acrA,	Zhang et al.^37^	F: CAACGATCGGACGGGTTTC R: TGGCGATGCCACCGTACT
acrB,	Zhang et al.^37^	F: AGTCGGTGTTCGCCGTTAAC R: CAAGGAAACGAACGCAATACC
acrF,	Zhang et al.^37^	F: GCGGCCAGGCACAAAA R: TACGCTCTTCCCACGGTTTC
acrR	Zhang et al.^37^	F: GCGCTGGAGACACGACAAC R: GCCTTGCTGCGAGAACAAA
adeA	Zhang et al.^37^	F: CAGTTCGAGCGCCTATTTCTG R: CGCCCTGACCGACCAAT
vanA	Cardak et al.^36^	F: GGGAAAACGACAATTGC R: GTACAATGCGGCCGTA
vanB	Cardak et al.^36^	F: ATGGGAAGCCGATAGTC R: GATTTCGTTCCTCGACC
mcr1	Rebelo et al.^39^	F: AGTCCGTTTGTTCTTGTGGC R: AGATCCTTGGTCTCGGCTTG
mcr2	Rebelo et al.^39^	F: CAAGTGTGTTGGTCGCAGTT R: TCTAGCCCGACAAGCATACC
mcr3	Rebelo et al.^39^	F: AAATAAAAATTGTTCCGCTTATG R: AATGGAGATCCCCGTTTTT
mcr4	Rebelo et al.^39^	F: TCACTTTCATCACTGCGTTG R: TTGGTCCATGACTACCAATG
mcr5	Rebelo et al.^39^	F: ATGCGGTTGTCTGCATTTATC R: TCATTGTGGTTGTCCTTTTCTG
rtxA	Abioye et al.^30^	F: CTGAATATGAGTGGGTGACTTACG R: GTGTATTGTTCGATATCCGCTACG
rtxC	Abioye et al.^30^	F: CGACGAAGATCATTGACGAC R: CATCGTCGTTATGTGGTTGC
toxR	Abioye et al.^30^	F: CCTTCGATCCCTAAGCAATAC R: AGGGTTAGCAACGATGCGTAAG
ompU	Abioye et al.^30^	F: ACGCTGACGGAATCAACCAAAG R: GCGGAAGTTTGGCTTGAAGTAG
ctxA	Abioye et al.^30^	F: CTCAGACGGGATTGTTAGGCACG R: TCTATCTCTGTAGCCCCTATTACG
ctxB	Abioye et al.^30^	F: GATACACATAATAGAATTAAGGATG R: GGTTGCTTCTCATCATCGAACCAC
vpi	Abioye et al.^30^	F: GCAATTTAGGGGCGCGACGT R: CCGCTCTTTCTTGATCTGGTAG
hylA	Abioye et al.^30^	F: GAGCCGGCATTCATCTGAAT R: CTCAGCGGGCTAATACGGTTTA
tdh	Abioye et al.^30^	F: GTAAAGGTCTCTGACTTTTGGAC R: TGGAATAGAACCTTCATCTTCACC
tlh	Abioye et al.^30^	F: AAAGCGGATTATGCAGAAGCACTG R: GCTACTTTCTAGCATTTTCTCTGC
vppC	Abioye et al.^30^	F: GTCGGATTCAGAGTCGGTATTTAG R: GTTCCACTGCCCACCAAACA
vgrG	Abioye et al.^30^	F: GAAGACGAAGCGAACCAAG R: ATTGAACCATCACTGTTCATCAC
hcp	Abioye et al.^30^	F: CAAGAAGCTCACGTTGATG R: TTGATTGAGTAGTAGTGCTCTTG
vcgC	Gxalo et al.^40^	F: AGCTGCCGATAGCGATCT R: CGCTTAGGATGATCGGTG
vcgE	Gxalo et al.^40^	F: CTCAATTGACAATGATCT R: CGCTTAGGATGATCGGTG

**Software and algorithms**		

APIWEB™ software	bioMerieux	https://apiweb.biomerieux.com/login
OriginPro 2025b	Origin Lab (USA), Origin 2025, Graphing and Analysis	https://www.originlab.com/2025
GraphPad Prism 9	GraphPad Software, Boston, USA	https://www.graphpad.com/updates/prism-900-release-notes
Mega 12	Molecular Evolutionary Genetic Analysis, version 12	https://www.megasoftware.net/
Basic Local Alignment Search Tool	NCBI Blast Analysis, Nucleotide Blast	https://blast.ncbi.nlm.nih.gov/Blast.cgi
Interactive Tree of Life (iTOL)	Ciccarelli FD et al., Science.; 2006	https://itol.embl.de/
ArcGIS 10.8 software	Geographical Information System ArcGIS based on Corine Land Cover (CLC)	https://desktop.arcgis.com/

### Method details

#### Study area

The study was conducted at three sites along the Arrone River, from its source to mouth, approximately 32 km long, in the Lazio region of Central Italy. During the monthly sampling campaign in the 2023 Italian bathing water season, RW and artificial plastic substrates (APSs) were sampled to examine pathogenic bacteria and their antibiotic resistance. To evaluate the potential anthropogenic impacts on the area, land-use analysis was also conducted using ArcGIS 10.8 software.

#### Design and Development of Artificial Plastic Substrate tool

The APSs sampling tool was designed based on our prototype,^9^ and employed in two parts as follows (Figure 1).

The mobilized part has been improved in terms of the number of polymers, parts, and shape. The most common plastic polymers found in aquatic environments were examined. Polystyrene (PS), Polyethene Terephthalate (PET), Polymethyl Methacrylate (PMMA), Expanded Polyvinyl Chloride (PVC expanded), Polycarbonate (PC), Polyvinyl Chloride (PVC), and Polypropylene (PP) (Leroy Merlin, Italy). Each polymer’s size and shape were standardized to expose the bacteria to the same area for colonization, specifically a 10 × 10 cm square on each slide. For the assembly, slides were stacked vertically, with layers separated by plastic spacers (1.5 cm width, 3 cm diameter). The seven polymers were manually cut, and a central hole was drilled. The structure was secured by passing a fishing line and a steel wire through the hole of each slide and spacer.

The immobilized part consists of a net with stones inside, used to ensure stability and anchoring at the sampling point, with a rope. This part of APSs remains fixed at the sampling site for the entire sampling campaign.

#### Sample collection

At each sampling site, a 1-liter sample of RW was collected in sterile flasks, and the removable parts of the APSs were also collected. The removable parts of the APSs were carefully replaced with new ones during sampling. The APSs had been submerged at approximately 50 cm for a month prior to bacterial colonization. All samples were pre-labeled for identification, immediately stored in a biological transport bag to prevent cross-contamination, and transported in a portable refrigerator at 4°C in the dark. Analyses were conducted within 24 h of collection. Using a field template, the operator recorded the site name, weather conditions, water and air temperature (°C), pH, salinity (ppt), electrical conductivity (mS/cm), and dissolved oxygen (%) with a multiparameter probe (YSI Instruments). Additional observations useful for further data analysis and interpretation were also recorded.

#### Isolation of strains

Targeted bacteria, ESBL-producing Enterobacteriaceae and *Vibrio* spp., were recovered from RW samples using modified membrane filtration methods.^41^ Briefly, a 100 mL aliquot of the sample was filtered through a 0.45 μm nitrocellulose membrane (Sartorius, Goettingen, Germany) using a vacuum pump. The filter was placed into 100 mL of Mueller-Hinton (MH) broth (Condalab, Madrid, Spain) and incubated at 37 ± 1°C for 24 h (Intercontinental Equipment, Anzio, Italy). Then, 10 μL of each culture was seminated using the spread-plate method onto both ESBL chromogenic agar supplemented with ESBL (Condalab, Madrid, Spain) and *Vibrio* chromogenic agar (Condalab, Madrid, Spain). The plates were incubated for 18–24 h at 37 ± 1°C.

While for the detection of targeted pathogens from APSs samples, an ‘ad hoc' protocol was developed, employing a modified Tricycle method.^12^ The first step involved removing organic material, such as algae and aquatic invertebrates, from the APSs. Using a sterile scraper, material from both sides of the plastic slides was collected, resuspended in 5 mL of MH broth, and incubated at 25 ± 1°C for 4–5 h. Ten microliters of each culture were then analyzed using a spread-plate method following incubation conditions described above. The results for all samples were reported for each targeted bacteria as either present or absent, based on the observation of specific phenotypic characteristics, including colony morphology, color, and halo zones, in accordance with the media manufacturer’s interpretive criteria.

A single, well-isolated colony from chromogenic agar was re-streaked onto Mueller–Hinton (MH) agar and incubated for 18–24 h at 37 ± 1°C to obtain a pure culture. A colony from each pure strain was then stored at −80 °C in cryoinstant beads for further analysis. All experiments were performed in triplicate, including negative controls, under sterile conditions in a laminar flow hood (AURA Biosafety Cabinet, Class 2).

#### Biochemical and molecular identification of isolates

A single cryobead of each strain was revived by streaking onto MH agar plates and incubating for 18–24 h at 37 ± 1°C. Subsequently, a single colony was subjected to biochemical identification using the API-20E and API-20NE kits (for the identification of ESBL-producing Enterobacteriaceae and *Vibrio* spp., respectively) according to the manufacturer’s instructions (bioMérieux, Grassina, Italy). Results were interpreted using the APIWEB software (bioMérieux; https://apiweb.biomerieux.com, accessed September 2024).

Additionally, for DNA extraction, a single revived colony was resuspended in 1 mL of MH broth and then centrifuged (Thermo Scientific, Massachusetts, United States) at 13000 rpm for 3 min, and DNA was extracted following the manufacturer’s protocols of the kit (GRISP, Porto, Portugal), and its quality and quantity were evaluated with a micro-UV-Vis spectrophotometer (LifeReal, Hangzhou, China). A 1000 bp fragment of the 16S rRNA coding the region of the V4–V5 hypervariable regions was selectively amplified with two universal primers, forward 5′-AGAGTTTGATCMTGGCTCAG-3′ and reverse 5′-TACGGYTACCTTGTTACGACTT-3’.^42^ The 16S rRNA gene was used for PCR amplification (Biometra GmbH, Göttingen, Germany). The final volume of the PCR mixture was 25 μL, comprising 12.5 μL of 1X GRISP PCR Fast Mastermix, 2 μL of each primer, 20 ng/μL of template DNA, and 6.5 μL of nuclease-free water. PCR reaction conditions were: 3 min for pre-denaturation at 95°C, followed by 30 cycles of denaturation at 95°C for 30 s, annealing at 55°C for 30 s, elongation at 72°C for 60 s, and a final extension at 72°C for 10 min.

From the PCR products, 3 μL of each reaction mixture were visualized by electrophoresis on a 1% agarose gel in 1X TBE (Tris Borate EDTA) buffer (100 mM Tris-HCl, 90 mM boric acid, 1 mM Na2EDTA) with a 1 kb DNA ladder. The gel was stained with 2 μL of 1X GelRed and ran at 90 V for 45 min. Before sequencing, unincorporated nucleotides and primers were removed from the PCR products using the PCR and Gel Band Purification Kit. The purified samples were then sent for Sanger sequencing to Microsynth Seqlab GmbH (Göttingen, Germany).

#### Antibiotic resistance profiling

##### Antibiotic susceptibility test and Multiple Antibiotic Resistance (MAR) index

The antibiotic susceptibility profile of the isolated strains against the panel was determined using the Kirby-Bauer method,^43^ with the following antibiotics (Condalab, Madrid, Spain): gentamicin (GEN 10 μg), kanamycin (KAN 30 μg), ciprofloxacin (CIP 5 μg), ceftazidime (CAZ 30 μg), cefoxitin (FOX 30 μg), imipenem (IMP 10 μg), erythromycin (ERY 10 μg), nalidixic acid (NA 10 μg), tetracycline (TET 30 μg), chloramphenicol (CAM 30 μg), and sulfamethoxazole (SMX 25 μg). Interpretation of results as Resistant (R), Sensitive (S), or Intermediate (I) was performed according to the manufacturer’s instructions, using CLSI breakpoints.^44^

Multiple Antibiotic Resistance (MAR) indices were then determined for each isolate by using the formula:

$$MAR\;Index=\frac{Number\;of\;antibiotics\;to\;which\;the\;isolate\;is\;resistant}{Total\;number\;of\;antibiotics\;tested\;}$$

Results were interpreted using established criteria: MAR values ≥0.2 indicate exposure to high antibiotic pressure from high-risk sources, whereas values <0.2 indicate low antibiotic exposure from low-risk sources.^45^

##### Detection of Antibiotic Resistance Genes (ARGs)

The 34 gene-specific primers (Table S1) were used to detect ARGs and class 1 integrons in the strains studied, using PCR amplification. The final volume of the PCR mix was 25 μL, containing 1 U of Taq DNA polymerase, 2.5 μL of 10X Taq buffer, 0.5 μL of 10 mM dNTPs, 1 μL of each primer (10 μM), 20 ng/μL of template DNA, and nuclease-free water to volume. PCR reaction conditions were pre-denaturation: at 95 °C for 5 min; 35 cycles of denaturation at 95 °C for 30s, annealing at gene-specific temperatures as specified in Table S1 for 30s, and extension at 72 °C for 45s; followed by a final extension at 72 °C for 10 min 3 μL of PCR product was analyzed by electrophoresis on a 1.5–2.0% agarose gel in 1× TBE buffer with 2 μLof 1X Gel Red, using a 100 bp DNA ladder, at 90 V for 45 min. Gel images were captured and analyzed using the VWR Smart Gel Documentation System (VWR International, Pennsylvania, USA).

#### Detection of virulent genes in Vibrio strains

Twenty ng/μL of genomic DNA extracted from *Vibrio* species was used as a template in the PCR reaction to detect virulence-associated factors, as described in Section detection of antibiotic resistant genes. The species-specific target gene panel and PCR conditions are listed in Table S2.

### Quantification and statistical analysis

Statistical analyses were performed using OriginPro 2025b (Version 10.25, OriginLab Corporation, Massachusetts, USA) and GraphPad Prism 9 (Version 9.3.0, GraphPad Software, Boston, USA).

For comparative analyses, categorical variables were assessed using Fisher’s exact tests (for small sample sizes) or Chi-square tests with Yates’ correction, and effect sizes were reported as Cramér’s V. Continuous variables were analyzed using Student’s *t*-tests (parametric). If a *p-value* > 0.05, the null hypothesis is supported, and the data are normally distributed. For normally distributed data, parametric tests (a two-sample *t* test and repeated-measures ANOVA) were performed; for data that did not meet normality, a non-parametric test (Kruskal-Wallis ANOVA) was used (Figure 2).

Hierarchical clustering and heatmap generation were performed on OriginPro 2025b, using Euclidean distance as the similarity metric and complete linkage as the clustering algorithm for both bacterial species and antibiotic resistance genes. Heatmaps were visualized with dendrograms along both axes to illustrate clustering relationships and patterns of association (Figure 3).

The partial 16S rRNA gene sequences obtained in this study were aligned using MEGA 12 (Molecular Evolutionary Genetic Analysis). Kimura’s two-parameter model (K2P) was used to determine the nucleotide substitution model that best fits the data. A nucleotide BLAST was conducted to search all sequence alignments against the NCBI database (ncbi.nlm.nih.gov/geo/query/blast.html). The sequences obtained in this study were further aligned using Muscle (Figure 4). Phylogenetic relationships were inferred using the Neighbor-Joining method and the Interactive Tree of Life (iTOL) platform (https://itol.embl.de/).
