# Supplementary material for: Artificial plastic substrate for tracking and exploring antimicrobial-resistant bacteria transmission pathways in aquatic ecosystems
Source: iScience. 2026 Oct 30;29(11):118096. doi: 10.1016/j.isci.2026.118096 (PMC13742847; doi:10.1016/j.isci.2026.118096)
Supplement: Supplementary file 1 [file mmc1.pdf]

iScience, Volume *29*

Supplemental information

Artificial plastic substrate for tracking and exploring antimicrobial-resistant bacteria transmission pathways in aquatic ecosystems

Ifra Ferheen, Eline Boelee, Filippo Chiudioni, Maria Rosa Loffredo, Laura Mancini, Marco Martuzzi, Marc B. Neumann, Kevin Portune, Sonja Wanke, and Stefania Marcheggiani

Supplementary Data

Table S1: Antibiotic-resistant genes tested, related to Figure 3a, 3b

Selected panel of the antibiotic resistance genes with their classes, possible location, function, and conventional PCR details (sequence, annealing temperature, product size)

Class of Antibiotic	Gene(s)	Possible Location	Function	Primer Sequence 5'-3'	Annealing T (°C)	Product size (bp)	Ref.
**Class 1 Integrons**	*intI1-V*	Plasmids, chromosomes, transposons	Genetic elements capturing and spreading resistance genes	**F:** TCATGGCTTGTTATGACTGT<br>**R:** GTAGGGCTTATTATGCACGC	58	variable	1,2
	intI-1			**F:** GGCTTCGTGATGCCTGCTT<br>**R:** CATTCCTGGCCGTGGTTCT	60	146	
**Beta-lactamases**	*bla_CTX-M*	Plasmids, chromosomes	ESBL, hydrolyzes extended-spectrum β-lactams	**F:** GGAGGCGTGACGGCTTTT<br>**R:** TTCAGTGCGATCCAGACGAA	60	63	
	bla_SHV		ESBL or narrow-spectrum β-lactamase	**F:** TCCCATGATGAGCACCTTTAAA<br>**R:** TTCGTCACCGGCATCCA	58	90	
	bla_TEM		ESBL or narrow-spectrum β-lactamase	**F:** AGCATCTTACGGATGGCATGA<br>**R:** TCCTCCGATCGTTGTCAGAAGT	60	101	
	bla_OXA-1		Narrow or extended-spectrum β-lactamase	**F:** GGCACCAGATTCAACTTTCAAG<br>**R:** GACCCCAAGTTTCCTGTAAGTG	56	564	3
	bla_IMP		Carbapenemase, hydrolyzes carbapenems	**F:** CATTCAAGGGCTTTCTTGCTGC<br>**R:** ACGACGGCATAGTCATTTGC	54	538	
	bla_VIM		Metallo-β-lactamase (carbapenemase)	**F:** TTGACACTCCATTTACDG<br>**R:** GATYGAGAATTAAGCCACYCT	50	139	
	bla_KPC	Plasmids	Metallo-β-lactamase (carbapenemase)	**F:** GATGGTGTTTGGTCGCATA<br>**R:** CGAATGCGCAGCACCAG	53	390	
	bla_OXA-48		Carbapenemase	**F:** GCTTGATCGCCCTCGATT<br>**R:** GATTTGCTCCGTGGCCGAAA	52	281	
	bla_NDM-1		Metallo-β-lactamase (carbapenemase)	**F:** GGTTTGGCGATCTGGTTTTC<br>**R:** CGGAATGGCTCATCACGATC	54	621	
Chloramphenicol resistance gene	*cmlA1*	Plasmids	Efflux pump conferring chloramphenicol resistance	**F:** TAGGAAGCATCGGAACGTTGAT<br>**R:** CAGACCGAGCACGACTGTTG	60	101	1,2
	cmx(A)			**F:** GCGATCGCCATCCTCTGT<br>**R:** TCGACACGGAGCCTTGGT		107	
Tetracycline resistance gene	*tetA*	Plasmids, chromosomes	Efflux pumps conferring tetracycline resistance	**F:** GCTGTTTGTTCTGCCGGAAA<br>**R:** GGTTAAGTTCCTTGAACGCAAACT		65	
	tetB			**F:** AGTGCGCTTTGGATGCTGTA<br>**R:** AGCCCCAGTAGCTCCTGTGA		63	
	tetM		Ribosomal protection proteins for tetracycline resistance	**F:** CATCATAGACACGCCAGGACATAT<br>**R:** CGCCATCTTTTGCAGAAATCA	58	101	
	tetW			**F:** ATGAACATTCCCACCGTTATCTTT<br>**R:** ATATCGGCGGAGAGCTTATCC	59	101	
Sulfonamide resistance gene	*sul1*	Class 1 integrons, plasmids	Target modification	**F:** CGCACCGGAAACATCGCTGCAC<br>**R:** TGAAGTTCCGCCGCAAGGCTCG	67	162	
	sul2	Plasmids		**F:** TCCGGTGGAGGCCGGTATCTGG<br>**R:** CGGGAATGCCATCTGCCTTGAG	65	190	
	sul3			**F:** TCCGTTCAGCGAATTGGTGCAG<br>**R:** TTCGTTCACGCCTTACACCAGC	63	127	
Multi-drug resistance (efflux pump genes)	*acrA,*	Chromosome	Components/regulators of Acr efflux system (multi-drug resistance)	**F:** CAACGATCGGACGGGTTTC<br>**R:** TGGCGATGCCACCGTACT	60	62	
	acrB,			**F:** AGTCGGTGTTCGCCGTTAAC<br>**R:** CAAGGAAACGAACGCAATACC		74	
	acrF,			**F:** GCGGCCAGGCACAAAA<br>**R:** TACGCTCTTCCCACGGTTTC		60	
	acrR			**F:** GCGCTGGAGACACGACAAC<br>**R:** GCCTTGCTGCGAGAACAAA		60	
	adeA	Chromosome, plasmid	Part of AdeABC efflux pump, multidrug resistance	**F:** CAGTTCGAGCGCCTATTTCTG<br>**R:** CGCCCTGACCGACCAAT	59	72	
Glycopeptide resistance gene	*vanA*	Transposons or plasmids	Encodes a glycopeptide resistance protein which alters the target of vancomycin	**F:** GGGAAAACGACAATTGC<br>**R:** GTACAATGCGGCCGTA	54	732	4
	vanB	Chromosome	Encodes a glycopeptide resistance protein to altered peptidoglycan synthesis	**F:** ATGGGAAGCCGATAGTC<br>**R:** GATTTCGTTCCTCGACC	50	635	
Colistin Resistance genes	*mcr1*	Plasmids	Encodes a phosphoethanolamine transferase that modifies the lipid A component of the bacterial outer membrane, reducing colistin binding	**F:** AGTCCGTTTGTTCTTGTGGC<br>**R:** AGATCCTTGGTCTCGGCTTG	58	320	5
	mcr2			**F:** CAAGTGTGTTGGTCGCAGTT<br>**R:** TCTAGCCCGACAAGCATACC		715	
	mcr3			**F:** AAATAAAAATTGTTCCGCTTATG<br>**R:** AATGGAGATCCCCGTTTTT		929	
	mcr4			**F:** TCACTTTCATCACTGCGTTG<br>**R:** TTGGTCCATGACTACCAATG		1116	
	mcr5			**F:** ATGCGGTTGTCTGCATTTATC<br>**R:** TCATTGTGGTTGTCCTTTTCTG		1644	

Table S2: Virulance genes of *Vibrio* spp. tested, related to Figure 4b,

Selected panel of the antibiotic resistance genes with their class, possible location, function, and conventional PCR details (sequence, annealing temperature, product size)

Gene	Gene Product	Species	Primer Sequence (5′→3′)	Product Size (bp)	PCR Conditions (°C)	Ref.
rtxA	repeat like toxin subunit A	*V. cholerae*	F: CTGAATATGAGTGGGTGACTTACG<br>R: GTGTATTGTTCGATATCCGCTACG	418	55	6
rtxC	repeat like toxin subunit C	*V. cholerae*	F: CGACGAAGATCATTGACGAC<br>R: CATCGTCGTTATGTGGTTGC	263	55	
toxR	transmembrane transcriptional activator	*V. cholerae*	F: CCTTCGATCCCTAAGCAATAC<br>R: AGGGTTAGCAACGATGCGTAAG	779	60	
ompU	pore-forming proteins of outer membrane	*V. cholerae*	F: ACGCTGACGGAATCAACCAAAG<br>R: GCGGAAGTTTGGCTTGAAGTAG	869	60	
ctxA	cholera toxin subunit A	*V. cholerae*	F: CTCAGACGGGATTGTTAGGCACG<br>R: TCTATCTCTGTAGCCCCTATTACG	301	60	
ctxB	repeat like toxin subunit B	*V. cholerae*	F: GATACACATAATAGAATTAAGGATG<br>R: GGTTGCTTCTCATCATCGAACCAC	461	55	
vpi	*Vibrio* pathogenicity island	*V. cholerae*	F: GCAATTTAGGGGCGCGACGT<br>R: CCGCTCTTTCTTGATCTGGTAG	680	52	
hylA	El Tor hemolysin	*V. cholerae*	F: GAGCCGGCATTCATCTGAAT<br>R: CTCAGCGGGCTAATACGGTTTA	481	60	
tdh	thermostable direct hemolysin	*V. parahaemolyticus*	F: GTAAAGGTCTCTGACTTTTGGAC<br>R: TGGAATAGAACCTTCATCTTCACC	270	55	6
tlh	thermolabile hemolysin	*V. parahaemolyticus*	F: AAAGCGGATTATGCAGAAGCACTG<br>R: GCTACTTTCTAGCATTTTCTCTGC	450	55	
vppC	metalloprotease gene	*V. parahaemolyticus*	F: GTCGGATTCAGAGTCGGTATTTAG<br>R: GTTCCACTGCCCACCAAACA	427	53	
vgrG	Valine-glycine repeat protein G	*V. parahaemolyticus*	F: GAAGACGAAGCGAACCAAG<br>R: ATTGAACCATCACTGTTCATCAC	533	54	
hcp	hemolysin-coregulated protein	*V. parahaemolyticus*	F: CAAGAAGCTCACGTTGATG<br>R: TTGATTGAGTAGTAGTGCTCTTG	248	54	
vcgC	variable cholera gene cluster	*V. vulnificus*	F: AGCTGCCGATAGCGATCT<br>R: CGCTTAGGATGATCGGTG	278	56	7
vcgE	variable cholera gene cluster	*V. vulnificus*	F: CTCAATTGACAATGATCT<br>R: CGCTTAGGATGATCGGTG	278	56	

References:

1. Ferheen, I., Spurio, R., and Marcheggiani, S. (2024). Vehicle transmission of antibiotic-resistant pathogens mediated by plastic debris in aquatic ecosystems. iScience 27, 110026. https://doi.org/10.1016/j.isci.2024.110026.

2. Zhang, Y., Lu, J., Wu, J., Wang, J., and Luo, Y. (2020). Potential risks of microplastics combined with superbugs: Enrichment of antibiotic resistant bacteria on the surface of microplastics in mariculture system. Ecotoxicol. Environ. Saf. 187, 109852. https://doi.org/10.1016/j.ecoenv.2019.109852.

3. Magiorakos, A.-P., Srinivasan, A., Carey, R.B., Carmeli, Y., Falagas, M.E., Giske, C.G., Harbarth, S., Hindler, J.F., Kahlmeter, G., Olsson-Liljequist, B., et al. (2012). Multidrug-resistant, extensively drug-resistant and pandrug-resistant bacteria: an international expert proposal for interim standard definitions for acquired resistance. Clin. Microbiol. Infect. 18, 268–281. https://doi.org/10.1111/j.1469-0691.2011.03570.x.

4. Çardak, M., Altuğ, G., Ay, M., and Erol, Ö. (2016). Distribution of antibiotic resistance and the presence of vancomycin-resistance genes (*vanA* and *vanB*) in *Enterobacteriaceae* isolated from the Sea of Marmara, the Canakkale Strait and the Istanbul Strait, Turkey. Oceanol. Hydrobiol. Stud. 45, 182–190. https://doi.org/10.1515/ohs-2016-0017.

5. Rebelo, A.R., Bortolaia, V., Kjeldgaard, J.S., Pedersen, S.K., Leekitcharoenphon, P., Hansen, I.M., Guerra, B., Malorny, B., Borowiak, M., Hammerl, J.A., et al. (2018). Multiplex PCR for detection of plasmid-mediated colistin resistance determinants, *mcr-1, mcr-2, mcr-3, mcr-4* and *mcr-5* for surveillance purposes. Euro Surveill. 23, 17-00672. https://doi.org/10.2807/1560-7917.ES.2018.23.6.17-00672.

6. Abioye, O.E., Osunla, C.A., Nontongana, N., and Okoh, A.I. (2023). Occurrence of virulence determinants in *Vibrio cholerae*, *Vibrio mimicus*, *Vibrio alginolyticus*, and *Vibrio parahaemolyticus* isolates from important water resources of Eastern Cape, South Africa. BMC Microbiol. 23, 316. https://doi.org/10.1186/s12866-023-03060-z.

7. Gxalo, O., Digban, T.O., Igere, B.E., Olapade, O.A., Okoh, A.I., and Nwodo, U.U. (2021). Virulence and Antibiotic Resistance Characteristics of *Vibrio* Isolates From Rustic Environmental Freshwaters. Front. Cell. Infect. Microbiol. 11, 732001. https://doi.org/10.3389/fcimb.2021.732001.
